# Tuning the cationic interface of simple polydiacetylene micelles to improve siRNA delivery at the cellular level†

**DOI:** 10.1039/c9na00571d

**Published:** 2019-09-24

**Authors:** Minh-Duc Hoang, Marie Vandamme, Gueorgui Kratassiouk, Guillaume Pinna, Edmond Gravel, Eric Doris

**Affiliations:** Service de Chimie Bioorganique et de Marquage (SCBM), CEA, Université Paris-Saclay 91191 Gif-sur-Yvette France edmond.gravel@cea.fr eric.doris@cea.fr; Plateforme ARN Interférence, Service de Biologie Intégrative et de Génétique Moléculaire (SBIGeM), I2BC, CEA, CNRS, Université Paris-Saclay 91191 Gif-sur-Yvette France guillaume.pinna@cea.fr

## Abstract

Polydiacetylene micelles were assembled from four different cationic amphiphiles and photopolymerized to reinforce their architecture. The produced micelles were systematically investigated, in interaction with siRNAs, for intracellular delivery of the silencing nucleic acids. The performances of the carrier systems were rationalized based on the cell penetrating properties of the micelles and the nature of their cationic complexing group, responsible for efficient siRNA binding and further endosomal escape.

## Abstract 2

Polydiacetylene micelles, bearing different cationic head groups, were systematically investigated for their ability to efficiently deliver functional siRNAs to cells.

## Graphical abstract

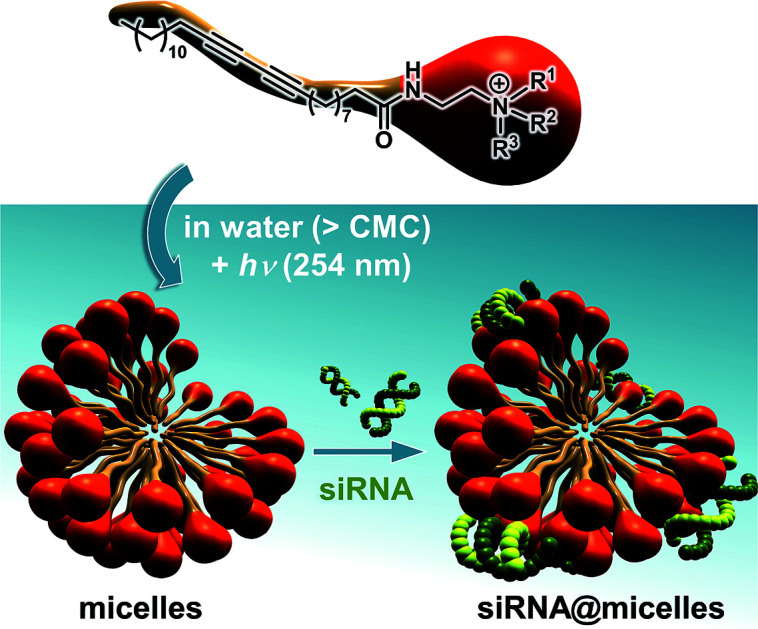

## Introduction

Since the discovery, two decades ago, of RNA interference,^1^ extensive research on small interfering RNAs (siRNAs) and their associated gene silencing properties has been carried out. Today, siRNA-mediated RNA interference is commonly used to study the function of genes but has also gained attention as a therapeutic option for the treatment of diseases such as cancer.^2^

As nucleic acids do not freely pass the cell plasma membrane, delivery of siRNA can be promoted using nanometric vectors. Taken as a whole, the efficacy of gene silencing is, for a large part, dependent on the cellular entry of siRNAs, also called transfection. This can be commonly achieved by complexing siRNAs with nanocarrier systems, providing protection to the siRNA and favoring cellular uptake/payload delivery.^3^ Transfection is a stepwise process which starts with the initial crossing of the plasma membrane through endocytosis and accumulation of the siRNA-loaded nanoparticles in endosomes/lysosomes from which they need to escape to reach the nucleus.^4^ The design of nanocarriers is thus of prime importance to maximize the amount of siRNA to be delivered into the cells. At the cellular level, examples of siRNA delivery systems include cationic polymers,^5^ lipids,^6^ peptides,^7^ carbon nanotubes,^8^ nanofibers^9^ and micelles.^10^ Although the latter systems are efficient and could behave in a synergistic fashion,^11^ there is a lack of understanding on how the chemical structures of the cationic region (responsible for electrostatic interactions with the nucleic acids) impacts the overall transfection process, and a rationale is yet to be formulated.

**Fig. 1:**
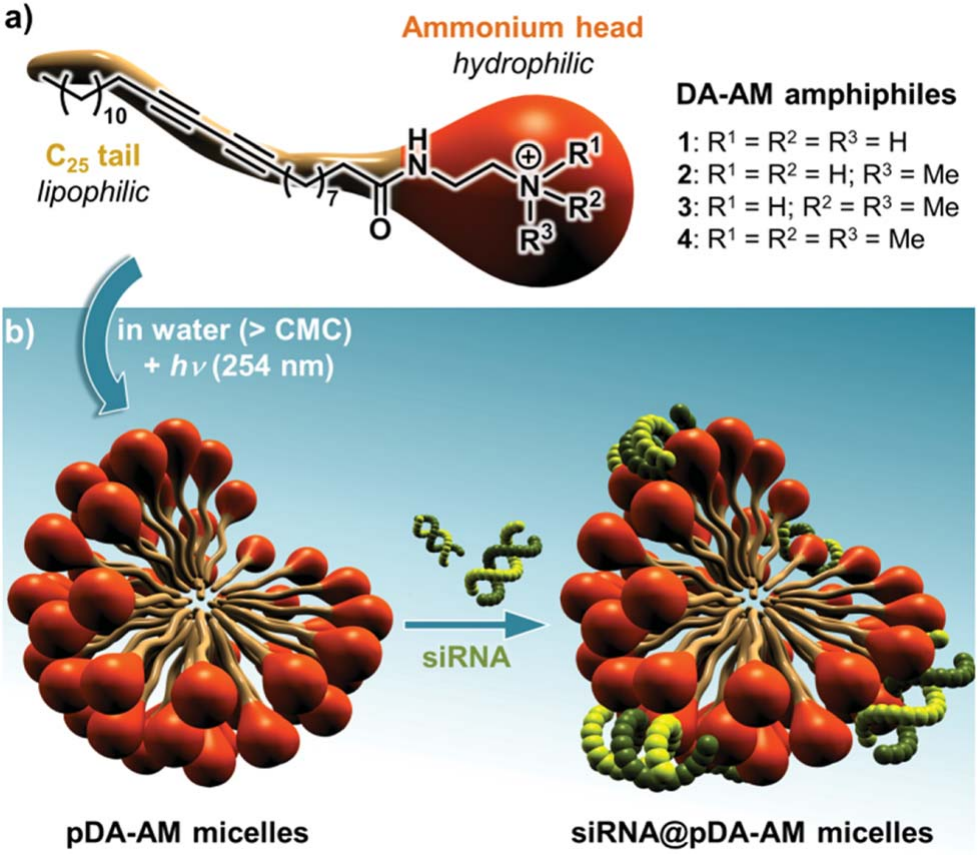
(a) Structure of the four DA-AM amphiphiles; (b) micelle assembly, photopolymerization and complexation with siRNA.

Our group has long been involved in the self-assembly of amphiphilic units into micelles for biomedical applications.^12^ For this study, a series of polydiacetylene micelles of identical structures, but incorporating different terminal ammonium groups, were assembled. The carrier systems were methodically studied with respect to transfection efficacy in order to better understand the role played by the substitution pattern of the complexing nitrogen atoms on the binding, transport, and release of the siRNA payload. The four micelle-forming amphiphiles discussed in this article are all based on the same *N*-(2-aminoethyl)pentacosa-10,12-diynamide backbone, yet incorporating a variable alkylation degree on the terminal amino-group, leading to primary (1), secondary (2), tertiary (3) and quaternary (4) ammoniums (Fig. 1a).

## Results and discussion

**Scheme 1:**
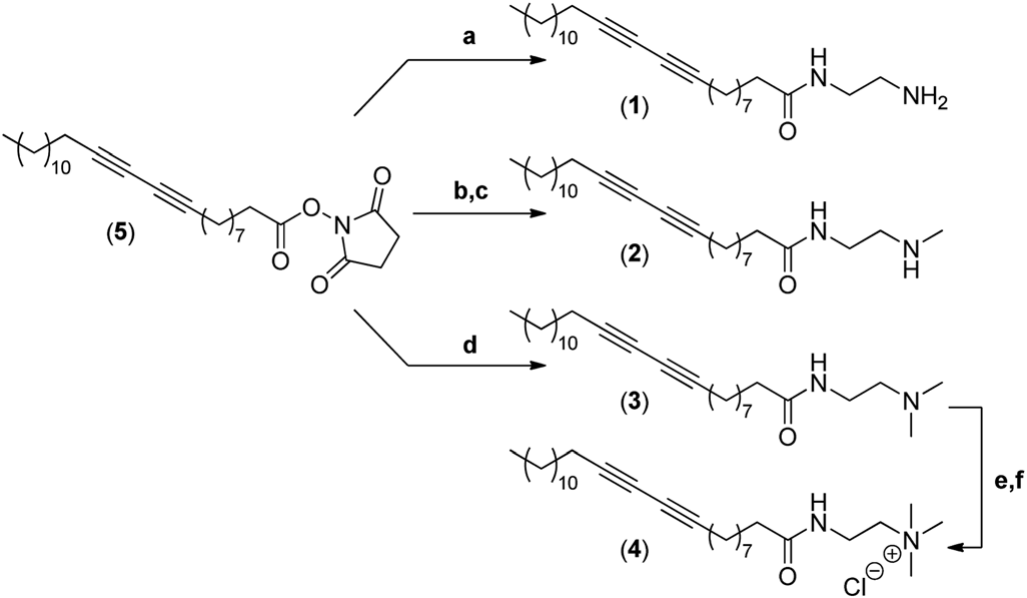
Synthesis of amphiphiles 1–4. (a) Ethylenediamine, CH_2_Cl_2_ (94%); (b) *N*-Boc-*N*-methylethylenediamine, CH_2_Cl_2_ (96%); (c) AcCl/MeOH (89%); (d) *N*,*N*-dimethylethane-1,2-diamine, CH_2_Cl_2_ (95%); (e) iodomethane (solv.) (88%); (f) Amberlyst A-26 resin (100%).

The synthesis of the four different micelle-forming diacetylene amphiphiles (DA-AM 1–4, see Fig. 1a for structures) was achieved starting from commercially available pentacosa-10,12-diynoic acid which was first treated with EDCI and NHS to afford activated ester 5 which is the common precursor to all the amphiphilic units to be prepared (Scheme 1). The reaction of 5 with ethylenediamine in excess led to the clean formation of the first amphiphile 1 (94% yield), bearing a terminal primary amine (–NH_2_).

Amphiphile 2, incorporating a terminal secondary amine (–NHMe), was obtained in 85% yield (over two steps) by the coupling of activated ester 5 with *N*-Boc-*N*-methylethylenediamine, followed by acidic deprotection of the methyl-amino group. Amphiphile 3 was produced in 95% yield from the reaction of 5 with *N*,*N*-dimethylethane-1,2-diamine, to give access to the amphiphile terminated by a tertiary amine (–NMe_2_).

The above amphiphile (3) also acted as a precursor to the synthesis of the quaternary ammonium 4 which was obtained by initial alkylation of 3 in methyl iodide followed by counter-ion exchange (I → Cl) over Amberlyst-26 resin. The quaternary ammonium chloride amphiphile 4 (–^+^NMe_3_) was isolated in 88% yield, over two steps.

The critical micelle concentration (CMC) of each of the newly synthesized amphiphiles was then measured by the pyrene encapsulation technique which indicated values of 0.06, 0.03, 0.03, and 0.01 mg mL^−1^ for DA-AM amphiphiles 1, 2, 3, and 4, respectively.

We next proceeded with the assembly of the corresponding micelles (Fig. 1b). Each amphiphile, in its ammonium form, was dispersed in a slightly acidic aqueous medium (10 mM HCl) at a concentration of 10 mg mL^−1^ ([c] > CMC) and was probe-sonicated for 30 min. The resulting clear suspension was then irradiated under UV (254 nm) for 5 h, producing a pale yellow colloid. The polymerization involved the formation of a ene-yne conjugated network through a topochemical 1,4-addition mechanism. The resulting pDA-AM micelles were further treated by dialysis for one week to remove non-polymerized amphiphilic units which are classically known to impart some cytotoxicity by cellular membrane destabilization.^13^ The polymerization step thus not only reinforced the stability of the colloidal micelles in highly dilute conditions (for example below the CMC), but also lowered their potential cytotoxicity, as dialyzed micelles interfered with HeLa cells proliferation/survival only at very high concentrations (Fig. S1†).

The micellar solution was then freeze dried and taken back in pure water before the pDA-AM micelles were characterized by Dynamic Light Scattering (DLS) analysis which indicated an average hydrodynamic diameter comprised between 6 and 9 nm, depending on the micelle type (Fig. S2†). Zeta potentials were also measured and all found to be of positive values (comprised between +15 and +30 mV). These results confirmed the overall cationic character of the micelles.

**Fig. 2:**
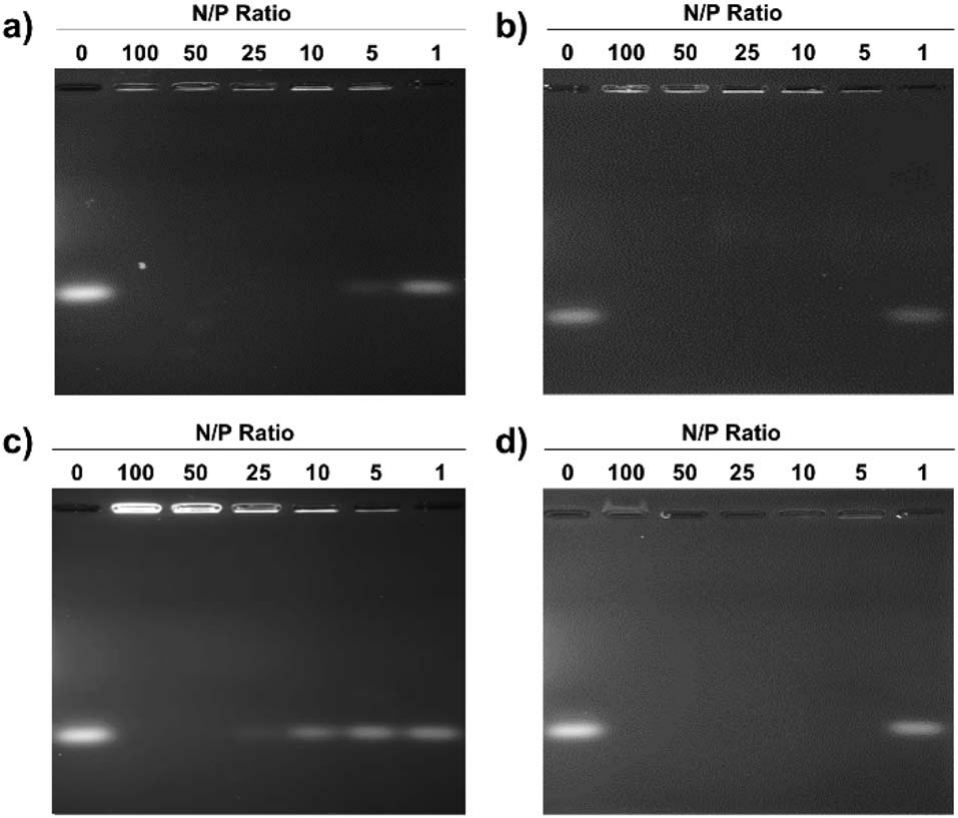
Agarose gel retardation assays show gradual complexation of siRNA by the pDA-AM micelles with increased nitrogen of micelle (N) to siRNA phosphate (P) ratio. (a) pDA-AM 1; (b) pDA-AM 2; (c) pDA-AM 3; and (d) pDA-AM 4.

Electrostatic adherence of siRNA to the cationic micelles, which is a prerequisite to transfection, was investigated by mixing increasing quantities of the pDA-AM 1–4 micelles to siRNA duplexes (20 pmol). Each sample was then analyzed by agarose gel electrophoresis, to separate free-migrating from micelle-bound siRNAs. As seen in Fig. 2, full retardation was observed at a ratio of micelle–nitrogen atoms (N) to siRNA phosphates (P) slightly above 5 for pDA-AM 1, *ca.* 5 for pDA-AM 2, slightly above 25 for pDA-AM 3, and *ca.* 5 for pDA-AM 4.

As the pDA-AM micelles were able to electrostatically bind siRNAs, we next evaluated their ability to efficiently deliver functional siRNAs to cells. To this end, we used a commercial pool of cytotoxic siRNAs specifically designed to target genes that are essential to cell proliferation/survival (AllStars Death Control, Qiagen). Transfection efficiency was assessed by measuring the cell proliferation/survival of HeLa cells, 72 h after transfection with the cytotoxic siRNA@micelle complexes. Control experiments were conducted using a “scrambled” siRNA sequence (UNR, negative control). siRNA@micelle complexes were tested at variable N/P ratios ranging from 5 to 50, while maintaining the siRNA concentration at 20 nM. In contrast to some of the previously described siRNA delivery procedures,^14^ in which cationic nanoparticles are transiently applied to cells in a medium without serum, we selected more stringent conditions by adding the siRNA@micelle complexes to cell culture medium containing serum. In the latter case, lipophilic components such as seric albumin could potentially interfere with the transfection process.

**Fig. 3:**
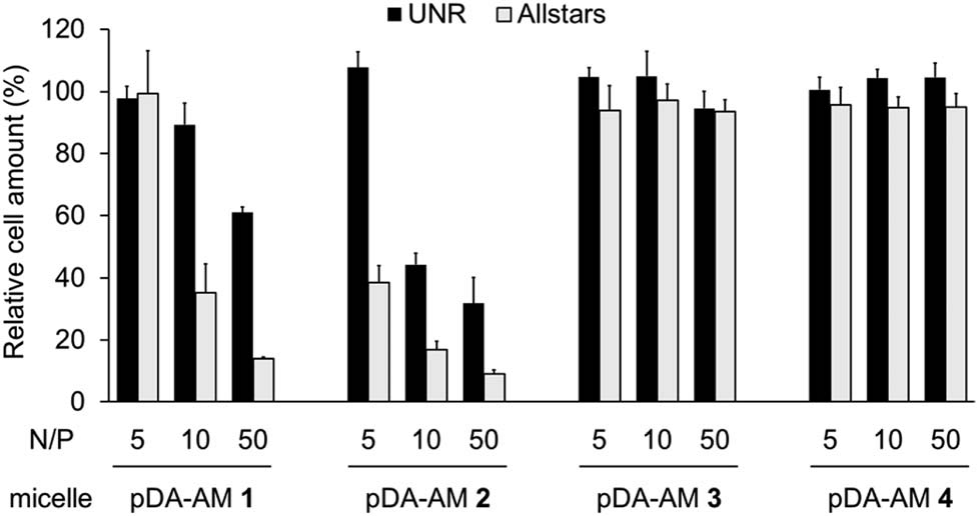
Transfection assays on HeLa cells using pDA-AM 1, pDA-AM 2, pDA-AM 3, and pDA-AM 4 micelles at different N/P ratios. siRNA concentration was kept at 20 nM for all experiments. UNR = negative control siRNA; AllStars = cytotoxic siRNA mixture.

Cell proliferation/survival was monitored upon cell fixation and counterstaining of cellular DNA by a fluorescent probe (Hoechst 33342), which allowed cell counting by High Content Imaging analysis. Data normalized to the “Untreated” control are reported in Fig. 3.

The first observation is that there is a strong effect of the cytotoxic siRNA pool when complexed with pDA-AM 1 and pDA-AM 2 micelles, starting at N/P ratios of 10 and 5, respectively. pDA-AM 2 micelles appeared to be the most potent in terms of transfection efficiency, whereas pDA-AM 3 and pDA-AM 4 micelles did not promote any transfection, whatever the N/P ratio considered. In fact, in the latter cases, we observed neither silencing effect nor intrinsic toxicity of the micelles. It is to be noted that some moderate unspecific cytotoxic (or -static) effect was associated to pDA-AM 1 and 2 micelles when in interaction with the negative control siRNA (UNR), especially at higher N/P ratios. Nevertheless, this effect is not detrimental *per se*, as strong silencing could already be detected for lower N/P ratios.

**Fig. 4:**
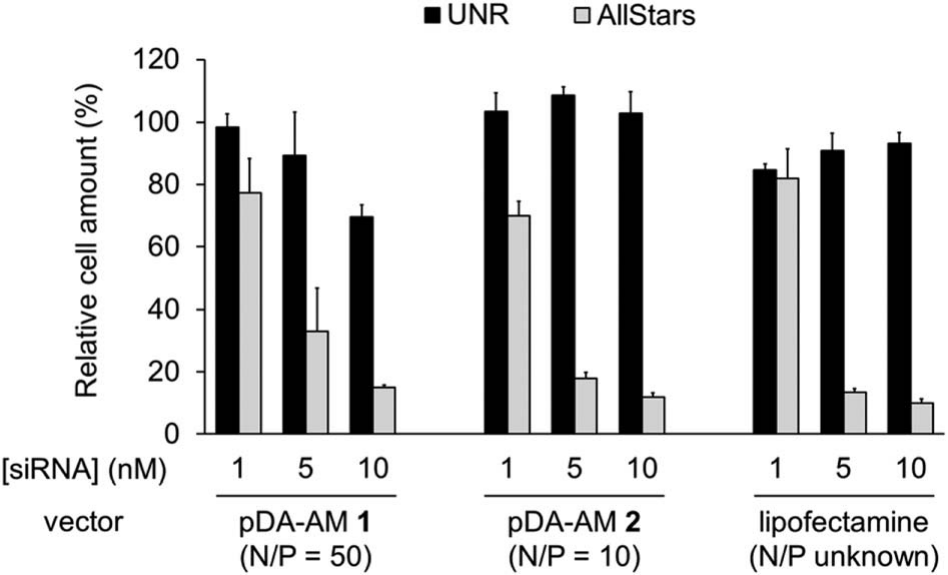
Transfection assays with pDA-AM 1 micelles at N/P = 50, pDA-AM 2 micelles at N/P = 10, and Lipofectamine (unknown N/P value). Final siRNA concentration = 1, 5, or 10 nM. UNR = negative control siRNA; AllStars = cytotoxic siRNA mixture.

The two micellar carriers that were found to be efficient for siRNA transfection (*i.e.* pDA-AM 1 and pDA-AM 2) were further tested at a fixed N/P ratio but with varying siRNA concentrations (from 1 to 10 nM), to determine the optimal transfection conditions, with minimal non-specific cytotoxicity (Fig. 4). A N/P ratio of 50 was selected for pDA-AM 1 micelles and transfection experiments were carried out with increasing amounts of the siRNA@pDA-AM 1 complex. The cytotoxic siRNA pool had a moderate effect on cell survival (80% cell survival) with 1 nM siRNA. Increasing the amount of siRNA@pDA-AM 1 complex to achieve a final siRNA concentration equal or above 5 nM led to a more pronounced effect, albeit with some associated minor side toxicity. Yet, when considering a N/P ratio fixed at 50, siRNA concentration of 10 nM appeared to be optimal for transfection, while maintaining non-specific cytotoxicity to an acceptable level.

Based on our initial experiments, we set the N/P ratio of pDA-AM 2 micelles at a value of 10. Under these conditions, 1 nM of siRNA led to a moderate, yet significant, cytotoxic effect (cell viability < 70%). Increasing the siRNA@pDA-AM 2 complex concentration up to 10 nM siRNA led to an enhancement of the transfection efficiency without any non-specific toxicity. Thus, pDA-AM 2 micelles at N/P = 10 can safely be used to efficiently transfect siRNAs down to 5 nM concentration. Performances of the cationic micelles were compared to that of a reference lipidic carrier system (Lipofectamine RNAi Max, Fig. 4). The results obtained with pDA-AM 1–2 were compared to that of Lipofectamine for which we observed a silencing effect in the same range as that of the micelles, upon complexation with 1, 5, or 10 nM of siRNA. Yet, the exact chemical structure of Lipofectamine RNAi max is unknown, thus preventing the calculation of the N/P ratio of the Lipofectamine/siRNA complex. This fact puts into perspective the relative effectiveness of the commercially available transfection agent.

It should be noted that, upon addition of the siRNA to the most active pDA-AM 2 micelle, we observed an increase of the hydrodynamic diameter of the micelles to *ca.* 80 nm (Fig. S3a†) and a decrease of the zeta potential value to 8 mV (N/P = 10) (Fig. S3b†). These observations are in line with the formation of the siRNA@micelle complex.

The results obtained in this study show that primary and secondary amines seem to be more efficient than their tertiary and quaternary counterparts when it comes to siRNA transfection. In the case of quaternary ammoniums, these findings are in agreement with common understanding of the siRNA release process. In fact, it is established that the so called “proton sponge effect” plays a central role in the siRNA escape from lysosomes inside of which the siRNA@micelle complexes are trapped.^15^ The phenomenon mostly relies on the buffering capacity of free amines, which induces an increase in lysosomal pH and influx of Cl^−^ ions. As a consequence, osmotic swelling induces disruption of the lysosome and release of siRNAs into the cytoplasm.^16^ This phenomenon cannot be operative in the case of per-methylated quaternary ammoniums (pDA-AM 4 micelles) as the amino groups borne by the micelle are no longer basic. On the contrary, the amino groups of pDA-AM 1–3 micelles are still likely to behave as proton sponges, and the cause of the lack of efficiency of pDA-AM 3 micelles has to be found elsewhere. We hypothesize that, in the case of pDA-AM 3, transfection is less active because of poor siRNA binding to the micelle, which was supported by the formation of the siRNA@micelle complex at only a high N/P ratio of *ca.* 25.

**Fig. 5:**
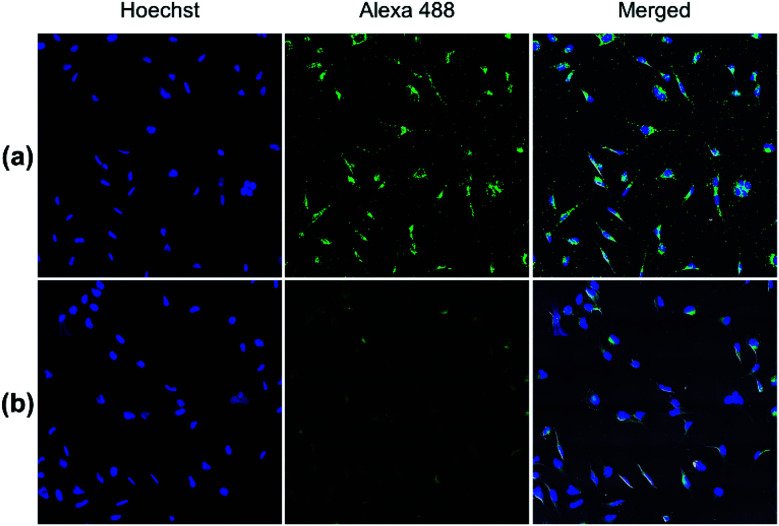
Confocal fluorescence images of (a) siRNA@pDA-AM 1 and (b) siRNA@pDA-AM 2 internalization in HeLa cells. pDA-AM 1 and pDA-AM 2 micelles were complexed with AllStars siRNA Alexa Fluor 488 (10 nM siRNA, N/P = 10) and nucleus was stained with Hoechst.

Both pDA-AM 1 and pDA-AM 2 micelles behaved in a very satisfactory fashion, with the highest transfection efficiency and negligible toxicity observed for pDA-AM 2 micelles. Optimization of the N/P ratio and tuning of the siRNA concentration allowed us to improve the delivery system in such a way that the siRNA achieved >80% inhibition of cell proliferation/survival, with no significant static effect of either the micelle alone or in combination with an irrelevant siRNA sequence. The difference in transfection efficiency between pDA-AM 1 and pDA-AM 2 micelles could lie in the higher basicity of the secondary amine of 2 where the methyl substituent adds stabilization to the cationic charge through polarization. It is likely that the buffering effect in the proton sponge phenomenon increases with basicity of the amine, leading to more efficient release of the siRNA payload into the cytoplasm, thus resulting in a higher silencing effect. Cellular internalization of the siRNA@pDA-AM 1 and siRNA@pDA-AM 2 complexes was further confirmed by confocal microscopy (Fig. 5) using a siRNA labeled with Alexa Fluor 488 fluorescent probe.

In fact, for the two active micelles (*i.e.* pDA-AM 1 and pDA-AM 2), we observed strong intracellular fluorescence signals at the vicinity of the nucleus. On the contrary, only weak signals were detected by confocal microscopy for the inactive micelles pDA-AM 3 and pDA-AM 4 (epifluorescence and confocal microscopy data are provided in the ESI for the four micelle–siRNA complexes, see Fig. S4 and S5,† respectively). These results suggest that, in addition to the siRNA binding/release properties of the micelles, cellular internalization also plays a central role in the overall efficacy of the transfection system.

## Material and methods

### General

Unless otherwise specified, chemicals were purchased from Sigma-Aldrich and used without further purification. CH_2_Cl_2_ was distilled from calcium hydride before use. ^1^H and ^13^C NMR spectra were recorded on a Bruker Avance DPX spectrometer operating at 400 and 100 MHz, respectively. Chemical shifts (*δ*) are given in ppm relative to the NMR solvent residual peak and coupling constants (*J*) in hertz. Mass spectra were recorded using a Mariner™ ESI-TOF spectrometer. Wavenumbers are given in cm^−1^ at their maximum intensity. Dynamic light scattering (DLS) measurements were carried out using a Vasco Flex instrument by Cordouan Technologies equipped with a laser diode (*λ* = 450 nm). Zeta potential measurements were performed on a Wallis instrument from Cordouan Technologies equipped with a laser diode (*λ* = 635 nm). For ultrasonic mixing, an ultrasonic probe (Branson Sonifier 450, Output 4, Duty cycle 30%) was used. Photo-polymerization experiments were carried out using a low pressure 40 W mercury UV lamp (Heraeus) at 254 nm.

### Synthesis of 2,5-dioxopyrrolidin-1-yl pentacosa-10,12-diynoate (5)

Under N_2_, to a solution of 10,12-pentacosadiynoic acid (2 g, 5.34 mmol, 1 equiv.) and *N*-hydroxysuccinimide (1.1 g, 1.8 equiv.) in CH_2_Cl_2_ (50 mL) were added 1-ethyl-(3-dimethylaminopropyl)carbodiimide hydrochloride (1.6 g, 1.5 equiv.). The mixture was stirred at room temperature overnight and then poured into water. The aqueous phase was extracted three times with CH_2_Cl_2_. The organic phases were collected, washed with brine, dried over MgSO_4_, filtered, and concentrated under vacuum. The crude product was purified by column chromatography (CH_2_Cl_2_) affording compound 5 (2.4 g, 96%).

^1^H NMR (400 MHz, CDCl_3_) *δ* 2.84 (s, 4H), 2.60 (t, *J* = 7.5 Hz, 2H), 2.24 (t, *J* = 6.9 Hz, 4H), 1.79–1.69 (m, 2H), 1.56–1.46 (m, 4H), 1.45–1.19 (m, 26H), 0.88 (t, *J* = 6.9 Hz, 3H) ppm.

^13^C NMR (100 MHz, CDCl_3_) *δ* 169.32, 168.78, 77.72, 77.55, 65.40, 65.31, 32.03, 31.03, 29.76, 29.74, 29.72, 29.59, 29.46, 29.21, 29.01, 28.97, 28.90, 28.81, 28.46, 28.38, 25.70, 24.64, 22.80, 19.31, 19.29, 14.24 ppm.

### Synthesis of *N*-(2-aminoethyl)pentacosa-10,12-diynamide (1)

Under N_2_, a solution of 5 (500 mg, 1.06 mmol, 1 equiv.) in CH_2_Cl_2_ (5 mL) was added dropwise to a solution of ethylene diamine (709 μL, 10 equiv.) diluted in 10 mL of CH_2_Cl_2_. The reaction mixture was stirred overnight at room temperature. The organic phase was washed with water and brine before it was dried over MgSO_4_, filtered, and concentrated under vacuum. The crude product was purified by column chromatography (CH_2_Cl_2_/MeOH/NH_4_OH, 90 : 10 : 1) affording compound 1 (417 mg, 94%).

^1^H NMR (400 MHz, CDCl_3_) *δ* 5.95 (s, 1H), 3.30 (q, *J* = 5.8 Hz, 2H), 2.83 (m, 2H), 2.25–2.16 (m, 6H), 1.62–1.25 (m, 32H), 0.87 (t, *J* = 6.9 Hz, 3H) ppm.

^13^C NMR (100 MHz, CDCl_3_) *δ* 173.58, 77.71, 77.56, 65.38, 65.31, 42.00, 41.54, 36.91, 32.02, 29.75, 29.73, 29.71, 29.58, 29.45, 29.34, 29.27, 29.20, 29.02, 28.96, 28.86, 28.45, 28.39, 25.85, 22.79, 19.30, 19.28, 14.23 ppm.

### Synthesis of *N*-(2-(methylamino)ethyl)pentacosa-10,12-diynamide (2)

#### Synthesis of *tert*-butylmethyl(2-(pentacosa-10,12-diynamido)ethyl)carbamate (BOC-protected 2)

Under N_2_, a solution of compound 5 (200 mg, 0.42 mmol, 1 equiv.) in CH_2_Cl_2_ (1 mL) was added dropwise to *N*-Boc-*N*-methylethylenediamine (111 mg, 1.5 equiv.) diluted in 5 mL of CH_2_Cl_2_. The reaction mixture was stirred overnight at room temperature. The organic phase was then washed with water and brine before it was dried over MgSO_4_, filtered, and concentrated under vacuum. The crude product was purified by column chromatography (CH_2_Cl_2_/AcOEt, 70 : 30) affording BOC-protected 2 (216 mg, 96%).

^1^H NMR (400 MHz, CDCl_3_) *δ* 6.38 (s, 1H), 3.47–3.28 (m, 4H), 2.86 (s, 3H), 2.28–2.06 (m, 6H), 1.64–1.12 (m, 41H), 0.86 (t, *J* = 6.9 Hz, 3H) ppm.

^13^C NMR (100 MHz, CDCl_3_) *δ* 173.79, 157.42, 80.12, 77.70, 77.55, 65.40, 65.33, 53.55, 47.46, 39.05, 36.88, 34.87, 32.03, 29.76, 29.74, 29.72, 29.59, 29.46, 29.39, 29.27, 29.21, 29.04, 28.97, 28.89, 28.52, 28.47, 28.42, 25.76, 22.80, 19.32, 19.30, 14.24 ppm.

#### Removal of the BOC-protecting group, completion of the synthesis of *N*-(2-(methylamino)ethyl)pentacosa-10,12-diynamide (2)

To a solution of the BOC-protected 2 (200 mg, 0.38 mmol) in 2 mL of MeOH was added, at 0 °C, acetyl chloride (536 μL, 20 equiv.). The reaction mixture was stirred at 0 °C for 1 h. The solvent was evaporated under reduced pressure and the crude product was dispersed and centrifuged three times (10 000×*g*, 3 min) in Et_2_O to afford compound 2 (156 mg, 89%).

^1^H NMR (400 MHz, CDCl_3_) *δ* 8.32 (s, 2H), 7.72–7.62 (m, 1H), 3.75–3.63 (m, 2H), 3.35–3.23 (m, 2H), 2.80 (t, *J* = 5.0 Hz, 3H), 2.39–2.28 (m, 2H), 2.23 (t, *J* = 7.0 Hz, 4H), 1.67–1.13 (m, 32H), 0.87 (t, *J* = 6.9 Hz, 3H) ppm.

^13^C NMR (100 MHz, CDCl_3_) *δ* 178.50, 77.80, 77.56, 65.41, 65.31, 49.96, 36.90, 36.09, 33.95, 32.05, 29.78, 29.76, 29.74, 29.61, 29.48, 29.23, 29.15, 29.09, 29.00, 28.94, 28.85, 28.49, 28.39, 25.55, 22.82, 19.32, 14.23 ppm.

### Synthesis of *N*-(2-(dimethylamino)ethyl)pentacosa-10,12-diynamide (3)

Under N_2_, a solution of compound 5 (500 mg, 1.06 mmol, 1 equiv.) in CH_2_Cl_2_ (5 mL) was added dropwise to *N*,*N*-dimethylethylenediamine (232 μL, 6 equiv.) diluted in 10 mL of CH_2_Cl_2_. The reaction mixture was stirred overnight at room temperature. The organic phase was then washed with water and brine before it was dried over MgSO_4_, filtered, and concentrated under vacuum. The crude product was purified by column chromatography (CH_2_Cl_2_/MeOH, 90 : 10) affording compound 3 (446 mg, 95%).

^1^H NMR (400 MHz, CDCl_3_) *δ* 6.11 (s, 1H), 3.39–3.25 (m, 2H), 2.42 (t, *J* = 6.0 Hz, 2H), 2.30–2.11 (m, 12H), 1.67–1.14 (m, 32H), 0.86 (t, *J* = 6.9 Hz, 3H) ppm.

^13^C NMR (100 MHz, CDCl_3_) *δ* 173.43, 77.70, 77.57, 65.39, 65.33, 58.05, 45.14, 36.80, 36.60, 32.03, 29.76, 29.74, 29.72, 29.59, 29.46, 29.35, 29.30, 29.22, 29.06, 28.97, 28.89, 28.46, 28.42, 25.84, 22.80, 19.32, 14.24 ppm.

### Synthesis of *N*,*N*,*N*-trimethyl-2-(pentacosa-10,12-diynamido)ethan-1-aminium chloride (4)

#### Synthesis of *N*,*N*,*N*-trimethyl-2-(pentacosa-10,12-diynamido)ethan-1-aminium iodide (compound 4 with an iodine counterion)

Under N_2_, compound 3 (400 mg, 0.9 mmol) was stirred at room temperature for 3 days in iodomethane in excess (5 mL). Iodomethane solvent was evaporated under reduced pressure and the crude product was purified by column chromatography (CH_2_Cl_2_/MeOH, 90 : 10) affording iodinated 4 (465 mg, 88%).

^1^H NMR (400 MHz, CDCl_3_) *δ* 7.71 (t, *J* = 5.5 Hz, 1H), 3.94–3.72 (m, 4H), 3.46 (s, 9H), 2.34–2.15 (m, 6H), 1.69–1.12 (m, 32H), 0.87 (t, *J* = 6.9 Hz, 3H) ppm.

^13^C NMR (100 MHz, CDCl_3_) *δ* 174.99, 77.75, 77.62, 65.61, 65.37, 65.32, 54.67, 36.51, 34.29, 31.99, 29.72, 29.70, 29.69, 29.56, 29.42, 29.34, 29.29, 29.18, 29.03, 28.95, 28.91, 28.45, 28.42, 25.53, 22.76, 19.29, 14.21 ppm.

#### Counterion exchange (I → Cl), completion of the synthesis of *N*,*N*,*N*-trimethyl-2-(pentacosa-10,12-diynamido)ethan-1-aminium chloride (4)

A 1 cm-diameter column was packed with 2.5 g of wet anion exchange Amberlyst A-26 resin (Cl form) and washed successively with water and with water–MeOH mixtures (25 mL of each solvent mixture) to reach pure MeOH. The above-prepared iodinated 4 (21 mg, 0.036 mmol) in 0.8 mL of methanol was then passed slowly through the resin and further eluted with 25 mL of MeOH. The solvent was evaporated to afford compound 4 (17.7 mg, 100%). ESI(−)-MS experiment qualitatively confirmed that iodine was no longer present in the sample.

^1^H NMR (400 MHz, CDCl_3_) *δ* 8.61 (t, *J* = 4.9 Hz, 1H), 3.91–3.64 (m, 4H), 3.40 (s, 9H), 2.31–2.14 (m, 6H), 1.65–1.12 (m, 32H), 0.85 (t, *J* = 6.9 Hz, 3H) ppm.

^13^C NMR (100 MHz, CDCl_3_) *δ* 174.91, 77.74, 77.61, 65.61, 65.37, 65.33, 54.18, 36.32, 34.37, 32.01, 29.74, 29.72, 29.71, 29.58, 29.44, 29.40, 29.35, 29.20, 29.06, 28.97, 28.93, 28.46, 28.43, 25.54, 22.78, 19.28, 14.23 ppm.

### Critical micelle concentration (CMC) measurements

CMC values were measured using the pyrene inclusion method. This method takes advantage of the environment specific fluorescence of the pyrene probe, as a detection of organized lipidic environment. A set of dilutions were prepared from 10 mM stock solutions of micelles ranging from 2 mM to 1 μM. 1 μL of 1 mM DMSO solution of pyrene was added to 1 mL of each sample and stirred vigorously for 2 h before fluorescence measurement.

Fluorescence spectra were recorded at 339 nm UV excitation wavelength at 5 nm band pass. The relative intensities at 373 nm and 384 nm were recorded. The ratios of the relative fluorescence intensities *I*_373nm_/*I*_384nm_ were plotted against log of mM concentrations. CMC is deduced from the inflexion point.

### Assembly and dialysis of the micelles

Amphiphile (10 mg) was first protonated in 1 mL of CH_2_Cl_2_/CH_3_OH 80 : 20 and 1 μL of HCl (37%). The solvent was evaporated under reduced pressure and a white solid was formed. The solid was then solubilized in 1 mL of 10 mM HCl and sonicated with an ultrasonic probe for 30 min. The solution was then subjected to UV irradiation at 254 nm for 5 h to yield a pale yellow solution of photo-polymerized product. Deionized water was added to replace the volume that was lost by evaporation during the photo-polymerization process. The dialyses were performed in 3000 MWCO dialysis membranes (Thermo Fisher) against a 1000 times larger volume of slightly acidic water (0.1% v/v of HCl 37%) over 7 days.

### Size measurements by dynamic light scattering (DLS)

Six acquisitions (60 s each) were performed on the colloid samples. Mean hydrodynamic diameter values of 6.6, 7.6, 9.2, and 7.6 nm were recorded for pDA-AM 1, pDA-AM 2, pDA-AM 3, and pDA-AM 4 micelles, respectively.

### Zeta potential

Samples of the micelle colloids (10 mg mL^−1^, 1 mL) were introduced in a dedicated cuvette equipped with an electrode and measurements were performed under 7.2 mV electric field. Zeta potential values of 29, 17, 15, and 30 mV were recorded for pDA-AM 1, pDA-AM 2, pDA-AM 3, and pDA-AM 4 micelles, respectively.

### siRNA transfection and proliferation/survival assay

The human cervical adenocarcinoma HeLa cell line (ATCC) was routinely grown in DMEM (Sigma) supplemented with 10% [v/v] Fetal Bovine Serum (PAA), Penicillin (100 UI mL^−1^, Sigma) and streptomycin (100 μg mL^−1^, Sigma). For transfection purpose, siRNAs diluted in OPTIMEM (Gibco) were complexed with either our micelles at various N/P ratios, or with Lipofectamine RNAiMAX (Life Technologies), in collagen-coated (Rat tail collagen, Sigma), clear bottom, black-walled 384-well culture plates (Greiner μClear plates, Cat# 781091). After 20 min of complexation, HeLa cells were seeded on top of the complexes (1000 cells per well; final [siRNA] = 1–20 nM), and incubated for three days at 37 °C and 5% CO_2_ in a humidified incubator (Forma Stericycle, Thermo). Plates were then fixed overnight with *para*-formaldehyde (4% [w/v] in PBS, Sigma), and nucleic acids were stained with Hoechst 33342 (2 μg mL^−1^, Sigma). After a PBS wash, plates were imaged on a High Content Imaging device (Operetta HCS epifluorescence microscope, PerkinElmer). Three fields per well were acquired at 10× magnification in the blue channel (*λ*_ex_ = 380 ± 20 nm; *λ*_em_ = 445 ± 35 nm). An automated algorithm was developed under Harmony 3.0 (PerkinElmer) as described before.^17^ Briefly, the algorithm segments nuclear Regions of Interest (ROI) based on the DNA-bound Hoechst fluorescence, then quantifies the total cell amount per well. All results were normalized to the untreated wells, and expressed as averaged results ± SD of four wells per condition. A pool of cytotoxic siRNAs (AllStars maximal death control, Qiagen) and a scrambled siRNA (UNR, target sequence: AAGCCGGTATGCCGGTTAAGT, Qiagen) were used as positive and negative phenotypic controls of transfection efficiencies, respectively.

### Gel retardation assay

siRNA/micelle complexes were prepared by mixing 20 pmol of scrambled (UNR) siRNA diluted in OptiMEM with varying amounts of micelles to achieve specific N/P ratios, ranging from 1 to 100. After 20 min of incubation, DNA loading buffer (Thermo Fisher) was added to the resulting complexes, and samples were loaded into a 2% (w/v) agarose gel containing 1 mM EDTA and 40 mM Tris acetate buffer pH 8.0 (TAE, Gibco). Sample migration was performed at 70 V for 80 min, and after electrophoresis, the gel was stained during 30 min with TAE buffer containing 0.5 μg mL^−1^ ethidium bromide (Sigma-Aldrich). After two subsequent washes in TAE buffer, the siRNA@micelle complexes were detected with an UV Transilluminator (Vilber Lourmat) at 254 nm.

### Confocal microscopy

HeLa cells were reverse transfected with siRNA@pDA-AM micelles in 16-well glass Chamber Slides (Labtek Cat#178599, Nunc) to achieve an N/P ratio of 10 and a final siRNA concentration of 10 nM. Two different siRNAs were transfected, either our scrambled negative control siRNA (UNR), or an irrelevant sequence coupled to Alexa 488 fluorophore (AllStars Neg. siRNA AF 488, Cat# 1027292, Qiagen). 12 h post transfection, cells were fixed for 20 min with *para*-formaldehyde (4% [w/v] in PBS), and nucleic acids were stained with Hoechst 33342 (2 μg mL^−1^). The Labtek media chambers were then removed, and the glass slides were mounted on coverslips using mounting medium (Shandon Immu-mount, Thermo). Image acquisition of fixed cells was performed with a Nikon A1 confocal microscope with a frame size of 512 × 512 pixels, using a ×20 (0.8 NA) objective, and Galvano lasers at 405 and 488 nm for illumination of Hoechst and Alexa 488 dyes, respectively. For quantification of a high number of cells, mosaics of 6 × 6 pictures were assembled with 15% stitching. Pictures acquired in the various fluorescence channels were then overlaid under ImageJ (Fiji).^18^

## Conclusions

Micelles, which differed by the nature of the cationic siRNA complexing units, were assembled and polymerized from the corresponding amine-terminated amphiphiles. Four different cationic micelles were produced bearing primary, secondary, tertiary and quaternary ammonium head groups, and evaluated with regards to their ability to bind siRNAs, enter cells, and induce endosomal escape. The micellar delivery system showed variable behavior depending on the cationic head and transfection performances were tentatively rationalized based on basicity (for “proton sponge”-related delivery issues), cellular internalization, or ability to interact with the nucleic acids (governing loading and transport properties). Two systems (primary and secondary amines) efficiently promoted siRNA delivery to HeLa cells albeit with some minor cytotoxicity at high N/P ratios. Yet, the latter effect could be avoided by adjusting both N/P ratio and siRNA concentration in such a way that secondary amine-micelles (pDA-AM 2) were shown to be fully operative at N/P = 10 with a fixed concentration of 5 nM siRNA. By simply tuning the cationic character of the polydiacetylene micelles, we were able to develop a promising carrier system which will further be investigated for *in vivo* silencing. In addition, our findings could also be useful for the optimization of known siRNA delivery vectors, by adjusting the fine balance between complexation stability and release capacity.

## Conflicts of interest

There are no conflicts to declare.

## Supplementary Material

NA-001-C9NA00571D-s001

## References

[cit1] Fire A., Xu S., Montgomery M. K., Kostas S. A., Driver S. E., Mello C. C. (1998). Nature.

[cit2] Draz M. S., Fang B. A., Zhang P., Hu Z., Gu S., Weng K. C., Gray J. W., Chen F. F. (2014). Theranostics.

[cit3] Tatiparti K., Sau S., Kashaw S. K., Iyer A. K. (2017). Nanomaterials.

[cit4] Dominska M., Dykxhoorn D. M. (2010). J. Cell Sci..

[cit5] Gary D. J., Puri N., Won Y. Y. (2007). J. Controlled Release.

[cit6] Schroeder A., Levins C. G., Cortez C., Langer R., Anderson D. G. (2009). J. Intern. Med..

[cit7] Boisguérin P., Deshayes S., Gait M. J., O'Donovan L., Godfrey C., Betts C. A., Wood M. J. A., Lebleu B. (2015). Adv. Drug Delivery Rev..

[cit8] Singh P., Samori C., Toma F. M., Bussy C., Nunes A., Al-Jamal K. T., Ménard-Moyon C., Prato M., Kostarelos K., Bianco A. (2011). J. Mater. Chem..

[cit9] Neuberg P., Hamaidi I., Danilin S., Ripoll M., Lindner V., Nothisen M., Wagner A., Kichler A., Massfelder T., Remy J. S. (2018). Nanoscale.

[cit10] Navarro G., Pan J., Torchilin V. P. (2015). Mol. Pharmaceutics.

[cit11] Zhu C., Jung S., Luo S., Meng F., Zhu X., Park T. G., Zhong Z. (2010). Biomaterials.

[cit12] Ogier J., Arnauld T., Carrot G., Lhumeau A., Delbos J.-M., Boursier C., Loreau O., Lefoulon F., Doris E. (2010). Org. Biomol. Chem..

[cit13] Neuberg P., Perino A., Morin-Picardat E., Anton N., Darwich Z., Weltin D., Mely Y., Klymchenko A. S., Remy J. S., Wagner A. (2015). Chem. Commun..

[cit14] Herrero M. A., Toma F. M., Al-Jamal K. T., Kostarelos K., Bianco A., Da Ros T., Bano F., Casalis L., Scoles G., Prato M. (2009). J. Am. Chem. Soc..

[cit15] Boussif O., Lezoualc'h F., Zanta M. A., Mergny M. D., Scherman D., Demeneix B., Behr J. P. (1995). Proc. Natl. Acad. Sci. U. S. A..

[cit16] Wojnilowicz M., Glab A., Bertucci A., Caruso F., Cavalieri F. (2019). ACS Nano.

[cit17] El helou R., Pinna G., Cabaud O., Wicinski J., Bhajun R., Guyon L., Rioualen C., Finetti P., Gros A., Mari B., Barbry P., Bertucci F., Bidaut G., Harel-Bellan A., Birnbaum D., Charafe-Jauffret E., Ginestier C. (2017). Cell. Reprogram..

[cit18] Schindelin J., Arganda-Carreras I., Frise E., Kaynig V., Longair M., Pietzsch T., Preibisch S., Rueden C., Saalfeld S., Schmid B., Tinevez J.-Y., White D. J., Hartenstein V., Eliceiri K., Tomancak P., Cardona A. (2012). Nat. Methods.

