# Supplementary material for: Tuning the cationic interface of simple polydiacetylene micelles to improve siRNA delivery at the cellular level
Source: Nanoscale Adv. 2019 Sep 24;1(11):4331–8. doi: 10.1039/c9na00571d (PMC9418740; doi:10.1039/c9na00571d)
Supplement: NA-001-C9NA00571D-s001 [file NA-001-C9NA00571D-s001.pdf]

Electronic Supplementary Material (ESI) for Nanoscale Advances.
This journal is © The Royal Society of Chemistry 2019

Supporting Information

Tuning the cationic interface of simple polydiacetylene micelles to improve siRNA delivery at the cellular level

Minh-Duc Hoang, Marie Vandamme, Gueorgui Kratassiouk, Guillaume Pinna, Edmond Gravel and Eric Doris

Table of contents

1- General **S2**
2- Chemical synthesis **S2**
2,5-dioxopyrrolidin-1-yl pentacosa-10,12-diynoate (**5**). S2
N-(2-aminoethyl)pentacosa-10,12-diynamide (**1**). S2
N-(2-(methylamino)ethyl)pentacosa-10,12-diynamide (**2**). S2
N-(2-(dimethylamino)ethyl)pentacosa-10,12-diynamide (**3**). S3
N,*N*,*N*-trimethyl-2-(pentacosa-10,12-diynamido) ethan-1-aminium chloride (**4**). S3
3- Characterization **S4**
Critical Micelle Concentration (CMC) measurements. S4
Assembly and dialysis of the micelles. S4
Size measurements by Dynamic Light Scattering (DLS). S4
Zeta potential. S4
4- Biology **S4**
siRNA transfection and proliferation/survival assay. S4
Gel retardation assay. S5
5- NMR spectra **S6**
2,5-dioxopyrrolidin-1-yl pentacosa-10,12-diynoate (**5**). S6
N-(2-aminoethyl)pentacosa-10,12-diynamide (**1**) S7
tert-butyl methyl(2-(pentacosa-10,12-diynamido)ethyl)carbamate S8
N-(2-(methylamino)ethyl)pentacosa-10,12-diynamide (**2**) S9
N-(2-(dimethylamino)ethyl)pentacosa-10,12-diynamide (**3**). S10
N,*N*,*N*-trimethyl-2-(pentacosa-10,12-diynamido)ethan-1-aminium iodide S11
N,*N*,*N*-trimethyl-2-(pentacosa-10,12-diynamido)ethan-1-aminium chloride (**4**) S12
6- Cytotoxicity studies of micelles **S13**
7- Dynamic Light Scattering of micelles **S13**
8- DLS and zeta potential of siRNA@pDA-AM 2 ..... **S14**
9- Epifluorescence images of transfected HeLa cells **S15**
10- Confocal Fluorescence images of transfected HeLa cells **S16**

1- General

Unless otherwise specified, chemicals were purchased from Sigma-Aldrich and used without further purification. CH_2Cl_2 was distilled from calcium hydride before use. ^1H and ^{13}C NMR spectra were recorded on a Bruker Avance DPX spectrometer operating at 400 and 100 MHz, respectively. Chemical shifts (δ) are given in ppm relative to the NMR solvent residual peak and coupling constants (J) in hertz. Mass spectra were recorded using a MarinerTM ESI-TOF spectrometer. Wavenumbers are given in cm^{-1} at their maximum intensity. Dynamic light scattering (DLS) measurements were carried out using a Vasco Flex instrument by Cordouan Technologies equipped with a laser diode (λ = 450 nm). Zeta potential measurements were performed on a Wallis instrument from Cordouan Technologies equipped with a laser diode (λ = 635 nm). For ultrasonic mixing, an ultrasonic probe (Branson Sonifier 450, Output 4, Duty cycle 30%) was used. Photo-polymerization experiments were carried out using a low pressure 40 W mercury UV lamp (Heraeus) at 254 nm.

2- Chemical synthesis

Synthesis of 2,5-dioxopyrrolidin-1-yl pentacosa-10,12-diynoate (5).

Under N_2, to a solution of 10,12-pentacosadiynoic acid (2 g, 5.34 mmol, 1 equiv.) and *N*-hydrosuccinimide (1.1 g, 1.8 equiv.) in CH_2Cl_2 (50 mL) were added 1-ethyl-(3-dimethylaminopropyl)carbodiimide hydrochloride (1.6 g, 1.5 equiv.). The mixture was stirred at room temperature overnight and then poured into water. The aqueous phase was extracted three times with CH_2Cl_2. The organic phases were collected, washed with brine, dried over $MgSO_4$, filtered, and concentrated under vacuum. The crude product was purified by column chromatography (CH_2Cl_2) affording compound **5** (2.4 g, 96%).
^1H NMR (400 MHz, $CDCl_3$) δ 2.84 (s, 4H), 2.60 (t, J = 7.5 Hz, 2H), 2.24 (t, J = 6.9 Hz, 4H), 1.79–1.69 (m, 2H), 1.56–1.46 (m, 4H), 1.45–1.19 (m, 26H), 0.88 (t, J = 6.9 Hz, 3H) ppm.
^{13}C NMR (100 MHz, $CDCl_3$) δ 169.32, 168.78, 77.72, 77.55, 65.40, 65.31, 32.03, 31.03, 29.76, 29.74, 29.72, 29.59, 29.46, 29.21, 29.01, 28.97, 28.90, 28.81, 28.46, 28.38, 25.70, 24.64, 22.80, 19.31, 19.29, 14.24 ppm.

Synthesis of *N*-(2-aminoethyl)pentacosa-10,12-diynamide (1).

Under N_2, a solution of **1** (500 mg, 1.06 mmol, 1 equiv.) in CH_2Cl_2 (5 mL) was added dropwise to a solution of ethylene diamine (709 µL, 10 equiv.) diluted in 10 mL of CH_2Cl_2. The reaction mixture was stirred overnight at room temperature. The organic phase was washed with water and brine before it was dried over $MgSO_4$, filtered, and concentrated under vacuum. The crude product was purified by column chromatography (CH_2Cl_2/MeOH/NH_4OH, 90:10:1) affording compound **1** (417 mg, 94%).

^1H NMR (400 MHz, $CDCl_3$) δ 5.95 (s, 1H), 3.30 (q, J = 5.8 Hz, 2H), 2.83 (m, 2H), 2.25 – 2.16 (m, 6H), 1.62 – 1.25 (m, 32H), 0.87 (t, J = 6.9 Hz, 3H) ppm.

^{13}C NMR (100 MHz, $CDCl_3$) δ 173.58, 77.71, 77.56, 65.38, 65.31, 42.00, 41.54, 36.91, 32.02, 29.75, 29.73, 29.71, 29.58, 29.45, 29.34, 29.27, 29.20, 29.02, 28.96, 28.86, 28.45, 28.39, 25.85, 22.79, 19.30, 19.28, 14.23 ppm.

Synthesis of *N*-(2-(methylamino)ethyl)pentacosa-10,12-diynamide (2).

*Synthesis of tert-butylmethyl(2-(pentacosa-10,12-diynamido) ethyl) carbamate (BOC-protected **2**):* Under N_2, a solution of compound **5** (200 mg, 0.42 mmol, 1 equiv.) in CH_2Cl_2 (1 mL) was added dropwise to *N*-Boc-*N*-methylethylenediamine (111 mg, 1.5 equiv.) diluted in 5 mL of CH_2Cl_2. The reaction mixture was stirred overnight at room temperature. The organic phase was then washed with water and brine before it was dried over $MgSO_4$, filtered, and concentrated under vacuum. The crude product was purified by column chromatography (CH_2Cl_2/AcOEt, 70:30) affording BOC-protected **2** (216 mg, 96%).

^{1}H NMR (400 MHz, $CDCl_3$) δ 6.38 (s, 1H), 3.47–3.28 (m, 4H), 2.86 (s, 3H), 2.28–2.06 (m, 6H), 1.64–1.12 (m, 41H), 0.86 (t, J = 6.9 Hz, 3H) ppm.

^{13}C NMR (100 MHz, $CDCl_3$) δ 173.79, 157.42, 80.12, 77.70, 77.55, 65.40, 65.33, 53.55, 47.46, 39.05, 36.88, 34.87, 32.03, 29.76, 29.74, 29.72, 29.59, 29.46, 29.39, 29.27, 29.21, 29.04, 28.97, 28.89, 28.52, 28.47, 28.42, 25.76, 22.80, 19.32, 19.30, 14.24 ppm.

*Removal of the BOC-protecting group, completion of the synthesis of N-(2-(methylamino)ethyl)pentacosa-10,12-diynamide (**2**):* To a solution of the BOC-protected **2** (200 mg, 0.38 mmol) in 2 mL of MeOH was added, at 0 °C, acetyl chloride (536 µL, 20 equiv.). The reaction mixture was stirred at 0°C for 1 h. The solvent was evaporated under reduced pressure and the crude product was dispersed and centrifuged three times (10 000 × *g*, 3 min) in Et_2O to afford compound **2** (156 mg, 89%).

^{1}H NMR (400 MHz, $CDCl_3$) δ 8.32 (s, 2H), 7.72–7.62 (m, 1H), 3.75–3.63 (m, 2H), 3.35–3.23 (m, 2H), 2.80 (t, J = 5.0 Hz, 3H), 2.39–2.28 (m, 2H), 2.23 (t, J = 7.0 Hz, 4H), 1.67–1.13 (m, 32H), 0.87 (t, J = 6.9 Hz, 3H) ppm.

^{13}C NMR (100 MHz, $CDCl_3$) δ 178.50, 77.80, 77.56, 65.41, 65.31, 49.96, 36.90, 36.09, 33.95, 32.05, 29.78, 29.76, 29.74, 29.61, 29.48, 29.23, 29.15, 29.09, 29.00, 28.94, 28.85, 28.49, 28.39, 25.55, 22.82, 19.32, 14.23 ppm.

Synthesis of *N*-(2-(dimethylamino)ethyl)pentacosa-10,12-diynamide (3).

Under N_2, a solution of compound **5** (500 mg, 1.06 mmol, 1 equiv.) in CH_2Cl_2 (5 mL) was added dropwise to *N,N*-dimethylethylenediamine (232 µL, 6 equiv.) diluted in 10 mL of CH_2Cl_2. The reaction mixture was stirred overnight at room temperature. The organic phase was then washed with water and brine before it was dried over $MgSO_4$, filtered, and concentrated under vacuum. The crude product was purified by column chromatography (CH_2Cl_2/MeOH, 90:10) affording compound **3** (446 mg, 95%).

^{1}H NMR (400 MHz, $CDCl_3$) δ 6.11 (s, 1H), 3.39–3.25 (m, 2H), 2.42 (t, J = 6.0 Hz, 2H), 2.30–2.11 (m, 12H), 1.67–1.14 (m, 32H), 0.86 (t, J = 6.9 Hz, 3H) ppm.

^{13}C NMR (100 MHz, $CDCl_3$) δ 173.43, 77.70, 77.57, 65.39, 65.33, 58.05, 45.14, 36.80, 36.60, 32.03, 29.76, 29.74, 29.72, 29.59, 29.46, 29.35, 29.30, 29.22, 29.06, 28.97, 28.89, 28.46, 28.42, 25.84, 22.80, 19.32, 14.24 ppm.

Synthesis of *N,N,N*-trimethyl-2-(pentacosa-10,12-diynamido) ethan-1-aminium chloride (4).

*Synthesis of N,N,N-trimethyl-2-(pentacosa-10,12-diynamido) ethan-1-aminium iodide (Compound **4** with an iodine counterion):* Under N_2, compound **3** (400 mg, 0.9 mmol) was stirred at room temperature for 3 days in iodomethane in excess (5 mL). Iodomethane solvent was evaporated under reduced pressure and the crude product was purified by column chromatography (CH_2Cl_2/MeOH, 90:10) affording iodinated **4** (465 mg, 88%).

^{1}H NMR (400 MHz, $CDCl_3$) δ 7.71 (t, J = 5.5 Hz, 1H), 3.94 – 3.72 (m, 4H), 3.46 (s, 9H), 2.34–2.15 (m, 6H), 1.69–1.12 (m, 32H), 0.87 (t, J = 6.9 Hz, 3H) ppm.

^{13}C NMR (100 MHz, $CDCl_3$) δ 174.99, 77.75, 77.62, 65.61, 65.37, 65.32, 54.67, 36.51, 34.29, 31.99, 29.72, 29.70, 29.69, 29.56, 29.42, 29.34, 29.29, 29.18, 29.03, 28.95, 28.91, 28.45, 28.42, 25.53, 22.76, 19.29, 14.21 ppm.

*Counterion exchange (I → Cl), completion of the synthesis of N,N,N-trimethyl-2-(pentacosa-10,12-diynamido) ethan-1-aminium chloride (**4**):* A 1 cm-diameter column was packed with 2.5 g of wet anion exchange Amberlyst A-26 resin (Cl form) and washed successively with water and with water-MeOH mixtures (25 mL of each solvent mixture) to reach pure MeOH. The above-prepared iodinated **4** (21 mg, 0.036 mmol) in 0.8 mL of methanol was then passed slowly through the resin and further eluted with 25 mL of MeOH. The solvent was evaporated to afford compound **4** (17.7 mg, 100%). ESI(−)-MS experiment qualitatively confirmed that iodine was no longer present in the sample.

^{1}H NMR (400 MHz, $CDCl_3$) δ 8.61 (t, J = 4.9 Hz, 1H), 3.91–3.64 (m, 4H), 3.40 (s, 9H), 2.31–2.14 (m, 6H), 1.65–1.12 (m, 32H), 0.85 (t, J = 6.9 Hz, 3H) ppm.

^{13}C NMR (100 MHz, $CDCl_3$) δ 174.91, 77.74, 77.61, 65.61, 65.37, 65.33, 54.18, 36.32, 34.37, 32.01, 29.74, 29.72, 29.71, 29.58, 29.44, 29.40, 29.35, 29.20, 29.06, 28.97, 28.93, 28.46, 28.43, 25.54, 22.78, 19.28, 14.23 ppm.

3- Characterization

Critical Micelle Concentration (CMC) measurements.

CMC values were measured using the pyrene inclusion method. This method takes advantage of the environment specific fluorescence of the pyrene probe, as a detection of organized lipidic environment. A set of dilutions were prepared from 10 mM stock solutions of micelles ranging from 2 mM to 1 µM. 1 µL of 1 mM DMSO solution of pyrene was added to 1 mL of each sample and stirred vigorously for 2 h before fluorescence measurement.
Fluorescence spectra were recorded at 339 nm UV excitation wavelength at 5 nm band pass. The relative intensities at 373 nm and 384 nm were recorded. The ratios of the relative fluorescence intensities I_{373nm}/I_{384nm} were plotted against log of mM concentrations. CMC is deduced from the inflexion point.

Assembly and dialysis of the micelles.

Amphiphile (10 mg) was first protonated in 1 mL of CH_2Cl_2/CH_3OH 80:20 and 1 µL of HCl (37%). The solvent was evaporated under reduced pressure and a white solid was formed. The solid was then solubilized in 1 mL of 10 mM HCl and sonicated with an ultrasonic probe for 30 min. The solution was then subjected to UV irradiation at 254 nm for 5 h to yield a pale yellow solution of photo-polymerized product. Deionized water was added to replace the volume that was lost by evaporation during the photo-polymerization process. The dialyses were performed in 3 000 MWCO dialysis membranes (Thermo Fisher) against a 1000 times larger volume of slightly acidic water (0.1% *v*/*v* of HCl 37%) over 7 days.

Size measurements by Dynamic Light Scattering (DLS).

Six acquisitions (60 s each) were performed on the colloid samples. Mean hydrodynamic diameter values of 6.6, 7.6, 9.2, and 7.6 nm were recorded for pDA-AM **1**, pDA-AM **2**, pDA-AM **3**, and pDA-AM **4** micelles, respectively.

Zeta potential.

Samples of the micelle colloids (10 mg mL^{-1}, 1 mL) were introduced in a dedicated cuvette equipped with an electrode and measurements were performed under 7.2 mV electric field. Zeta potential values of 29, 17, 15, and 30 mV were recorded for pDA-AM **1**, pDA-AM **2**, pDA-AM **3**, and pDA-AM **4** micelles, respectively.

4- Biology

siRNA transfection and proliferation/survival assay.

The human cervical adenocarcinoma Hela cell line (ATCC) was routinely grown in DMEM (Sigma) supplemented with 10% [*v*/*v*] Fetal Bovine Serum (PAA), Penicillin (100 UI mL^{-1}, Sigma) and Streptomycin (100 µg mL^{-1}, Sigma). For transfection purpose, siRNAs diluted in OPTIMEM (Gibco) were complexed with either our micelles at various N/P ratios, or with Lipofectamine RNAiMAX (Life Technologies), in collagen-coated (Rat tail collagen, Sigma), clear bottom, black-walled 384-well culture plates (Greiner µClear plates, Cat# 781091). After 20 min of

complexation, Hela cells were seeded on top of the complexes (1000 cells/well; final [siRNA] = 20 nM), and incubated for three days at 37 °C and 5% CO_2 in a humidified incubator (Forma Stericycle, Thermo). Plates were then fixed overnight with para-formaldehyde (4% [*w*/*v*] in PBS, Sigma), and nucleic acids were stained with Hoechst 33342 (2 µg mL^{-1}, Sigma). After a PBS wash, plates were imaged on a High Content Imaging device (Operetta HCS epifluorescence microscope, Perkin Elmer). Three fields per well were acquired at 10× magnification in the blue channel (λ_{ex} = 380 ± 20 nm; λ_{em} = 445 ± 35 nm). An automated algorithm was developed under Harmony 3.0 (Perkin Elmer) as described before.[17] Briefly, the algorithm segments nuclear Regions of Interest (ROI) based on the DNA-bound Hoechst fluorescence, then quantifies the total cell amount per well. All results were normalized to the untreated wells, and expressed as averaged results ± SD of four wells per condition. A pool of cytotoxic siRNAs (Allstars maximal death control, Qiagen) and a scrambled siRNA (UNR, target sequence: AAGCCGGTATGCCGGTTAAGT, Qiagen) were used as positive and negative phenotypic controls of transfection efficiencies, respectively.

Gel retardation assay.

siRNA/micelle complexes were prepared by mixing 20 pmol of scrambled (UNR) siRNA diluted in OptiMEM with varying amounts of micelles to achieve specific N/P ratios, ranging from 1 to 100. After 20 min of incubation, DNA loading buffer (Thermo Fisher) was added to the resulting complexes, and samples were loaded into a 2% (*w*/*v*) agarose gel containing 1 mM EDTA and 40 mM Tris acetate buffer pH 8.0 (TAE, Gibco). Sample migration was performed at 70 V for 80 min, and after electrophoresis, the gel was stained during 30 min with TAE buffer containing 0.5 µg mL^{-1} ethidium bromide (Sigma-aldrich). After two subsequent washes in TAE buffer, the siRNA@micelle complexes were detected with an UV Transilluminator (Vilber Lourmat) at 254 nm.

5- NMR spectra

2,5-dioxopyrrolidin-1-yl pentacosa-10,12-diynoate (5)

^{1}H-NMR

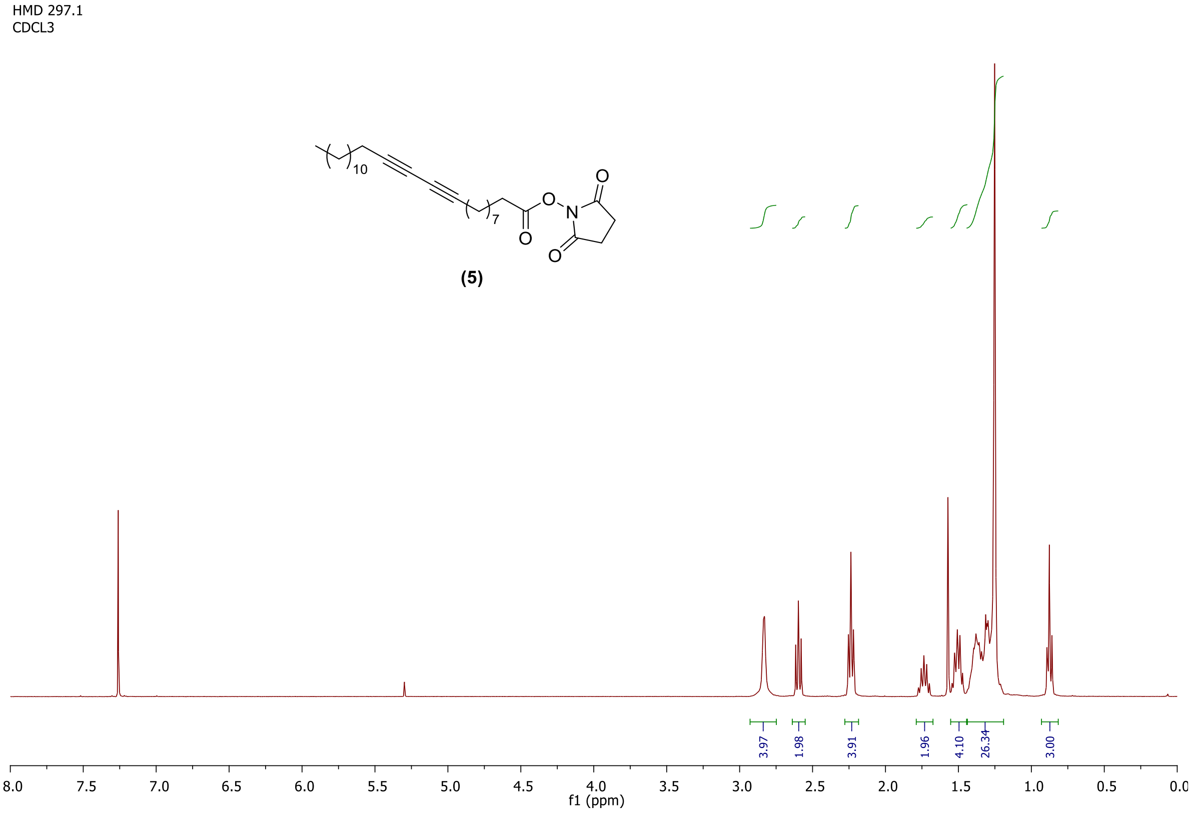

^{13}C-NMR

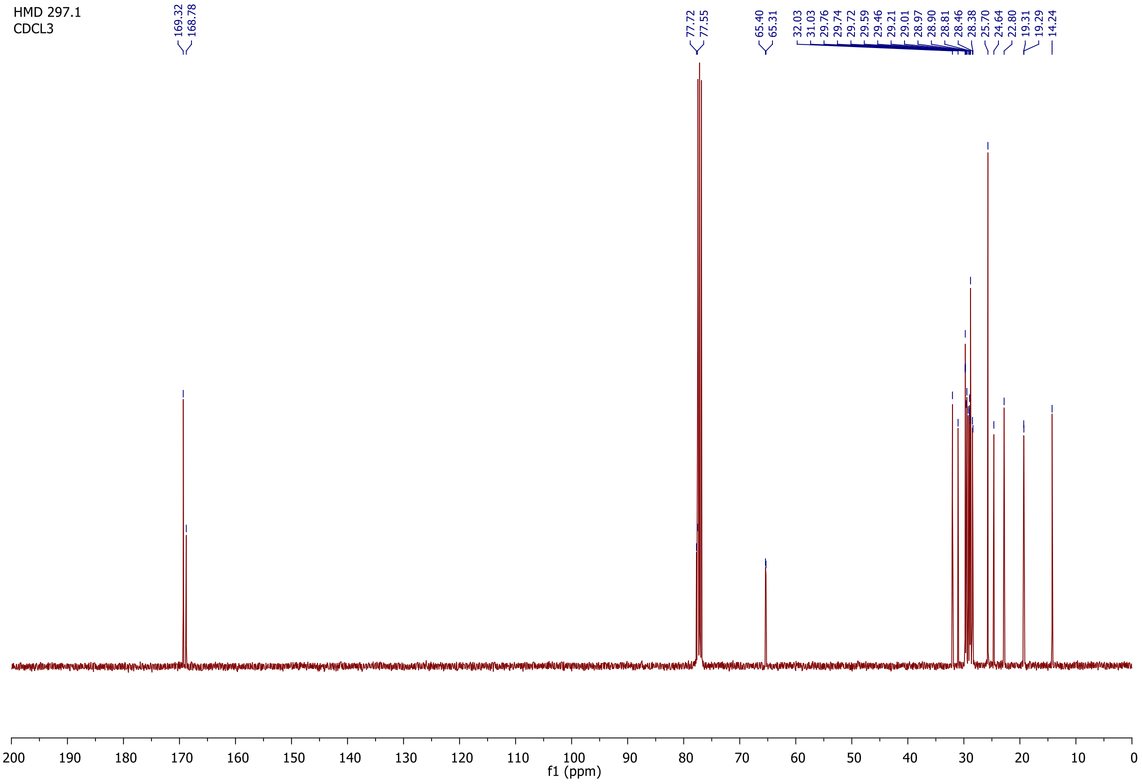

***N*-(2-aminoethyl)pentacosa-10,12-diynamide (1)**

^{1}H-NMR

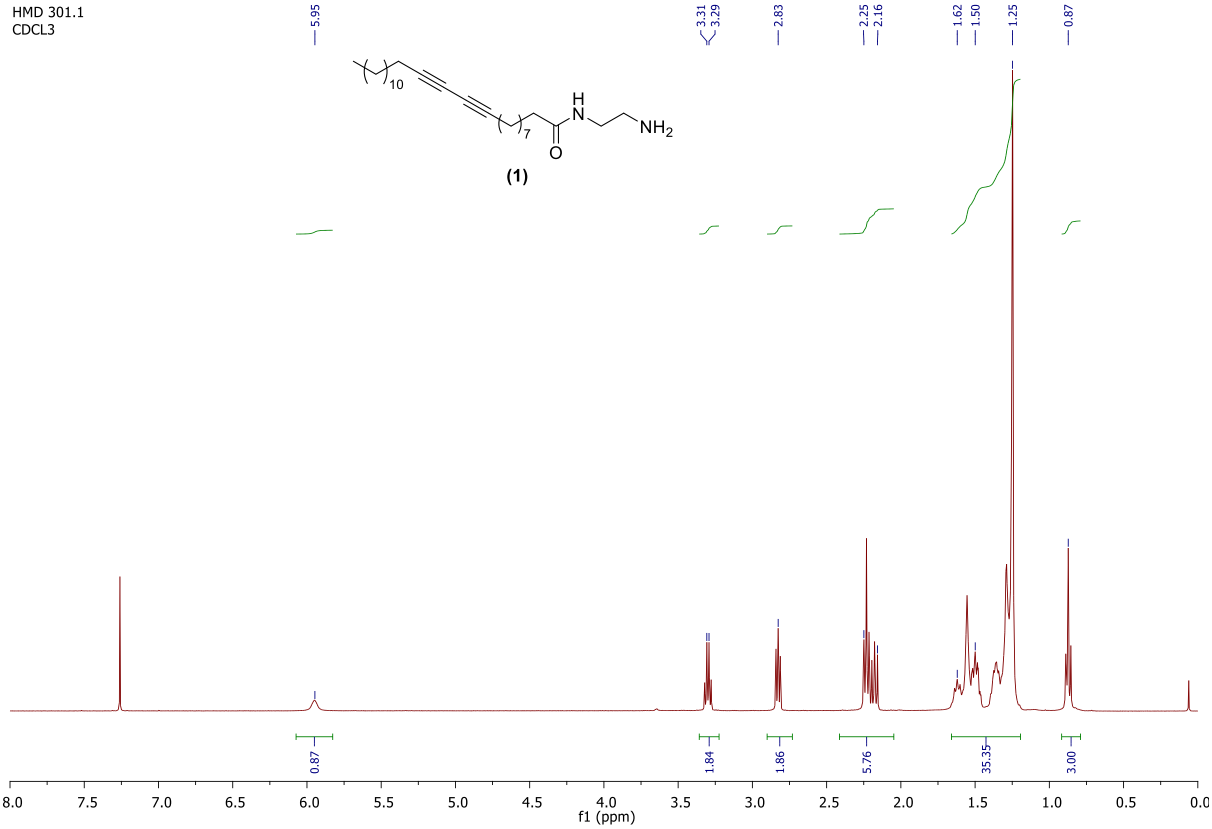

^{13}C-NMR

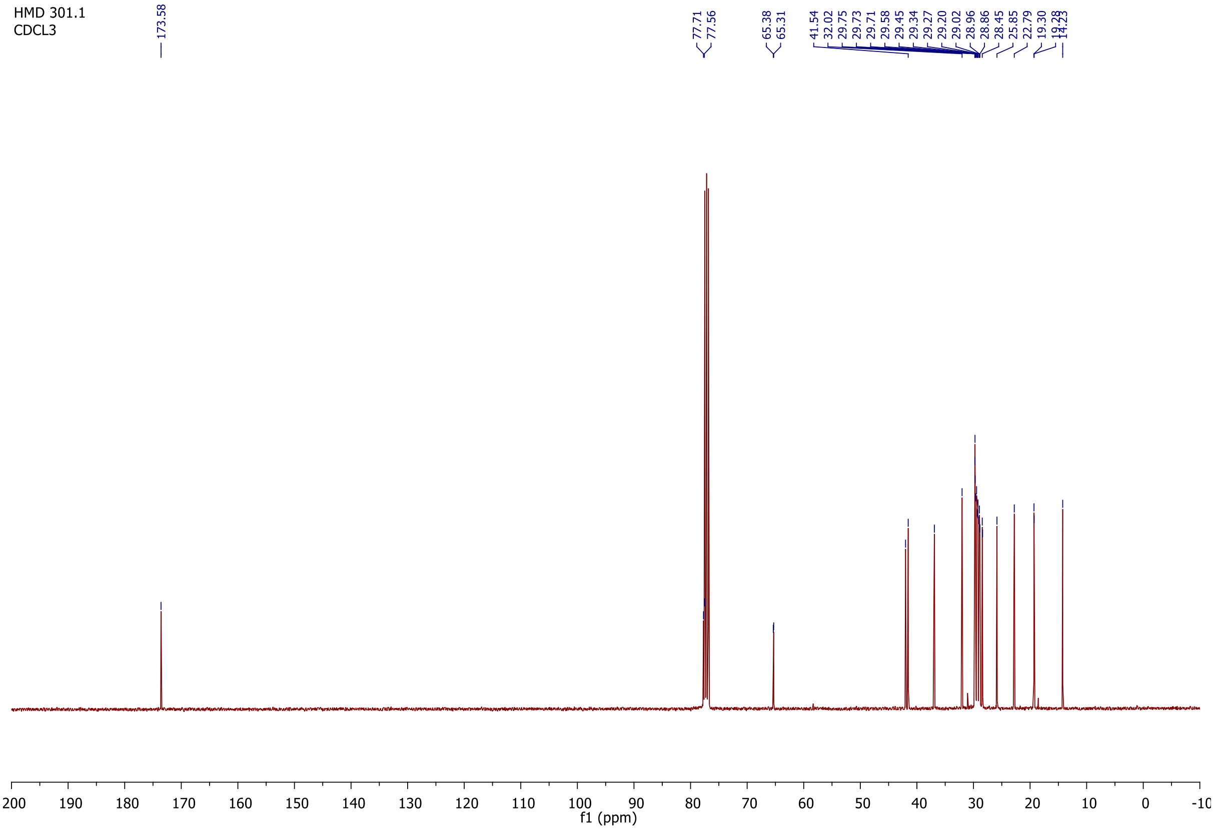

tert-butyl methyl(2-(pentacosa-10,12-diynamido)ethyl)carbamate

^{1}H-NMR

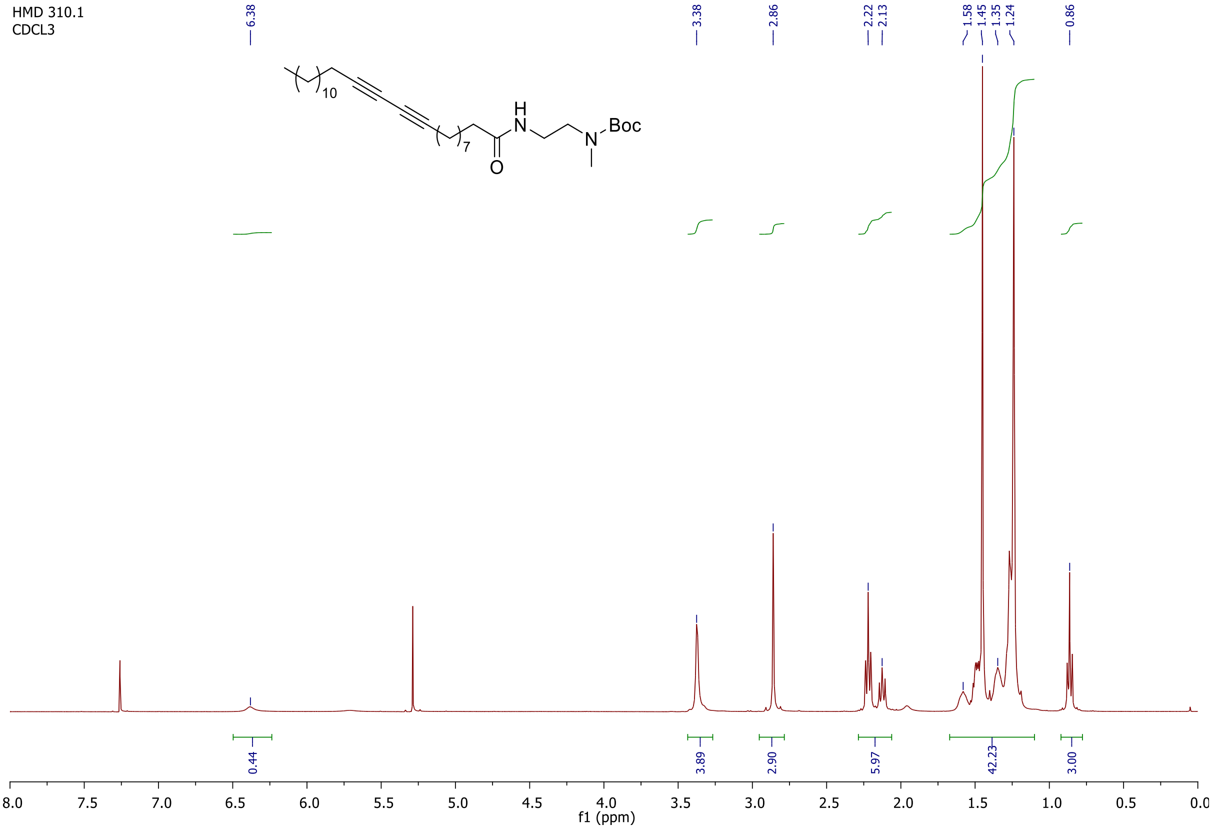

^{13}C-NMR

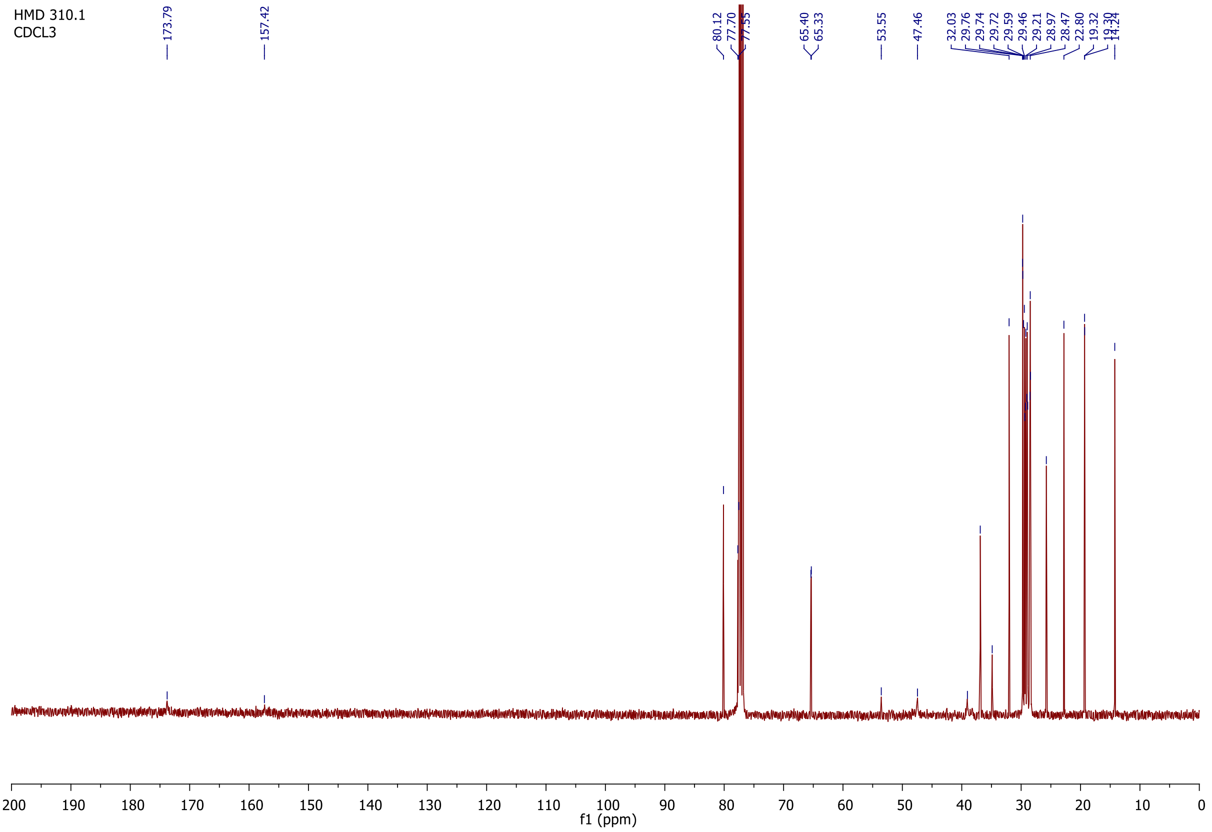

N-(2-(methylamino)ethyl)pentacosa-10,12-diynamide (2)

^{1}H-NMR

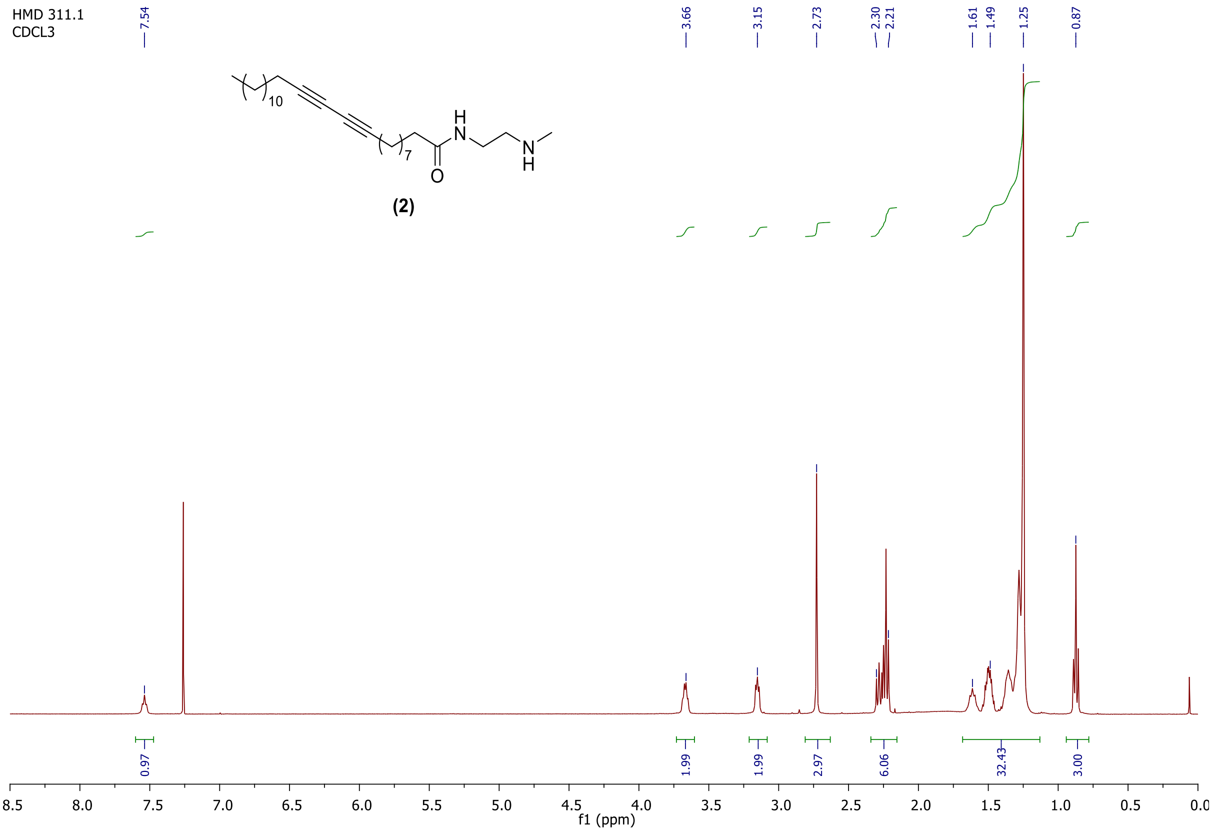

^{13}C-NMR

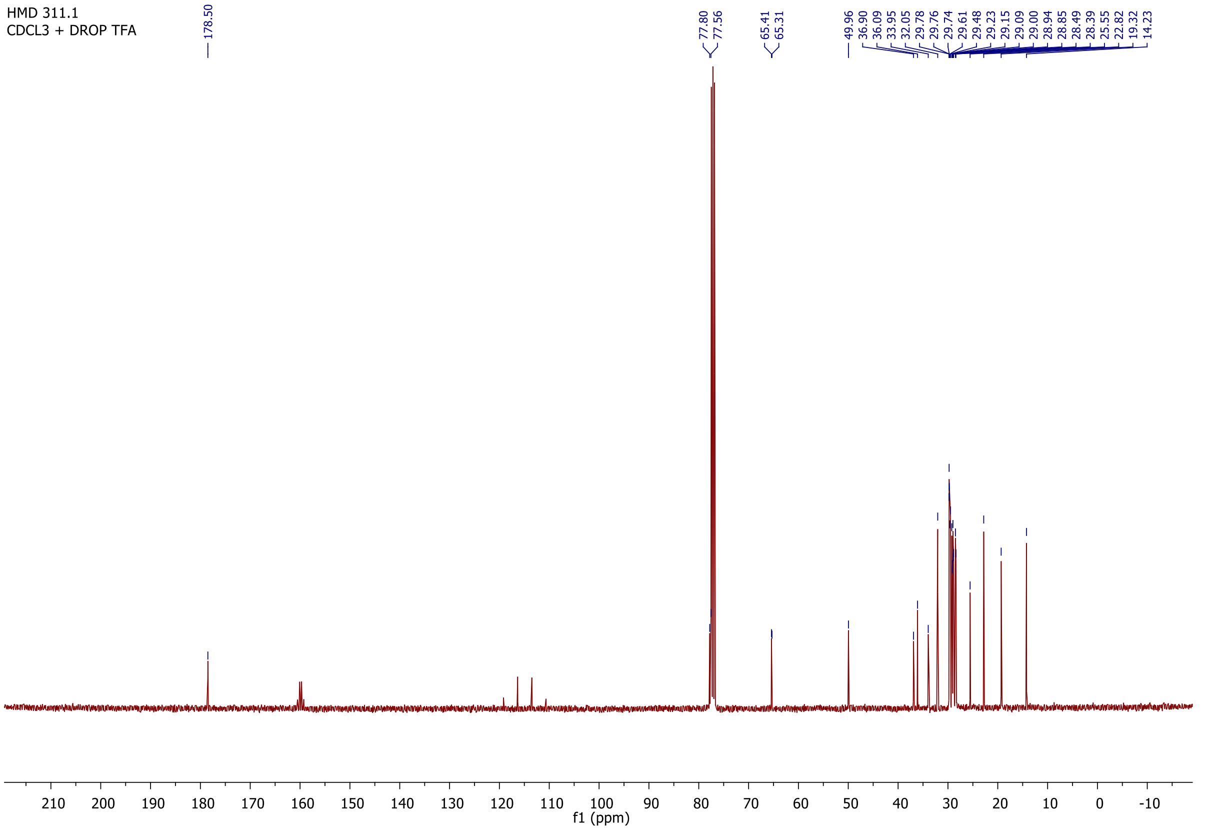

N-(2-(dimethylamino)ethyl)pentacosa-10,12-diynamide (3)

^{1}H-NMR

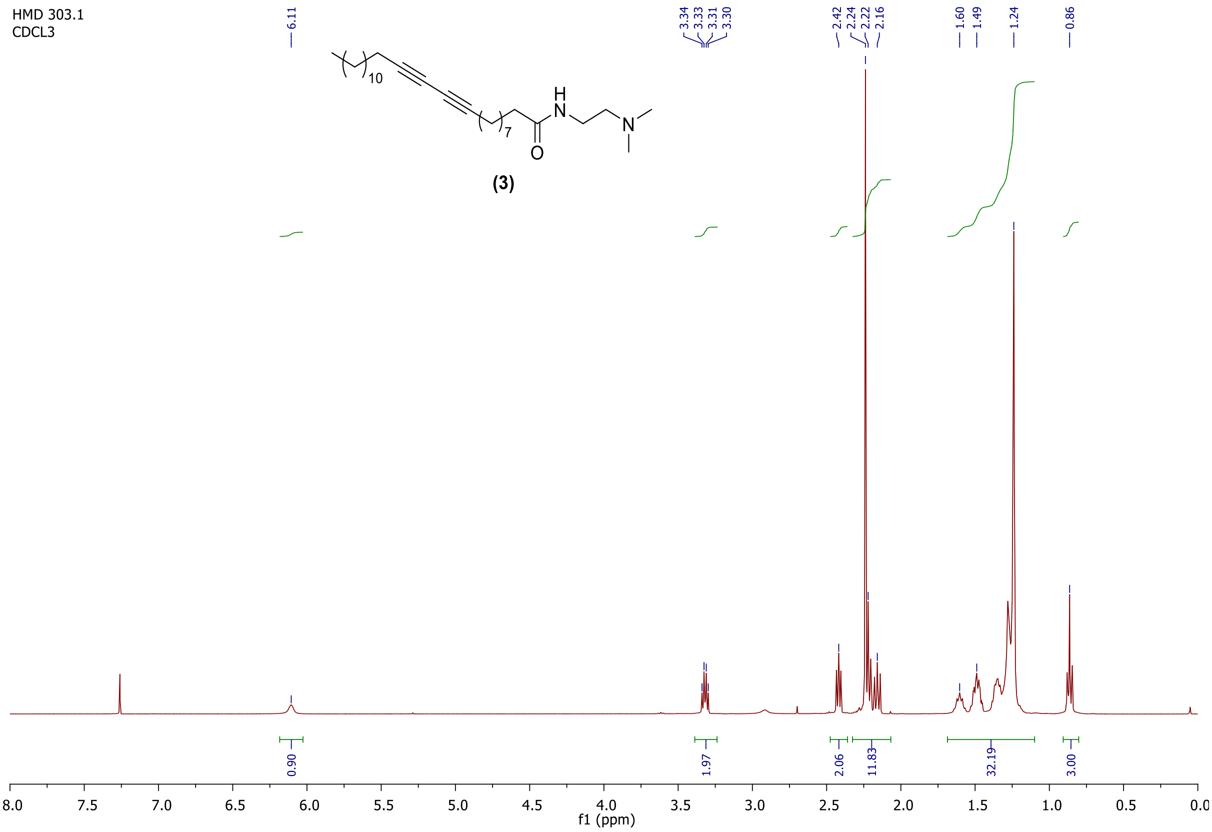

^{13}C-NMR

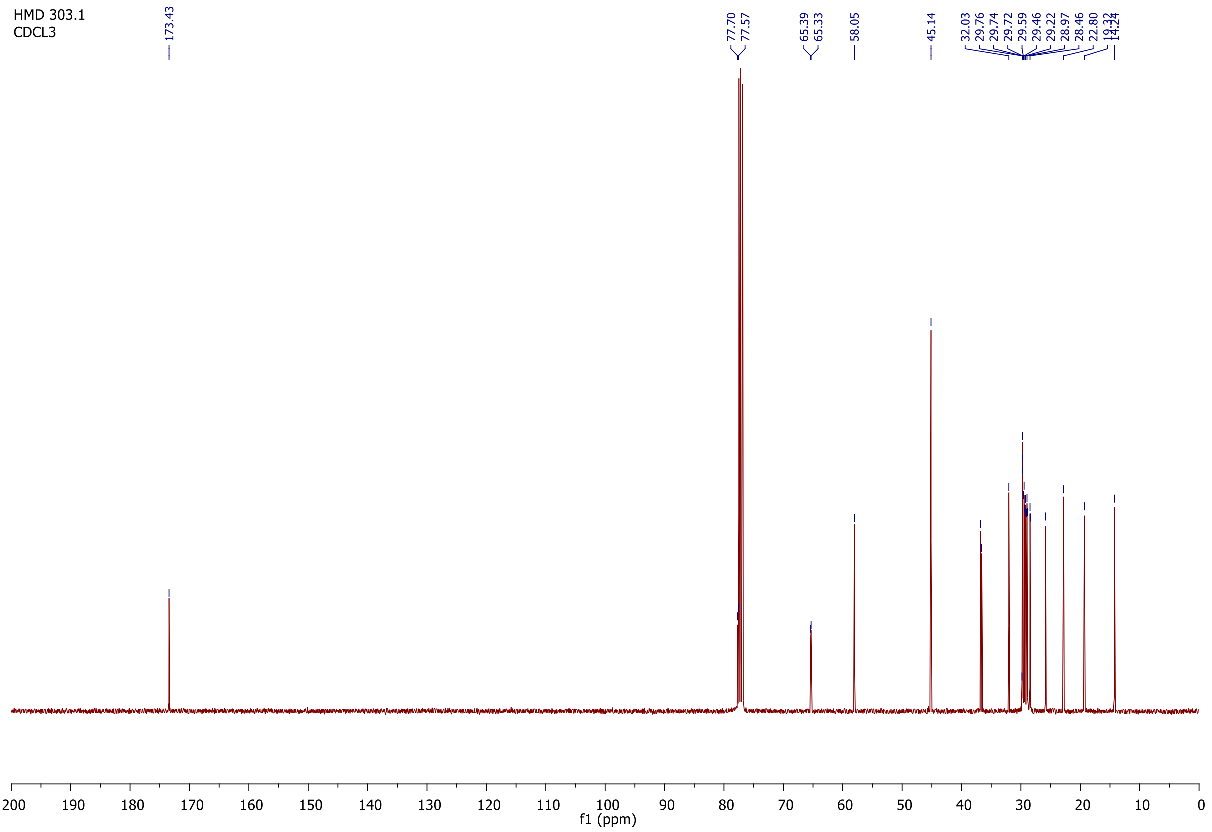

***N*,*N*,*N*-trimethyl-2-(pentacosa-10,12-diynamido)ethan-1-aminium iodide**

^{1}H-NMR

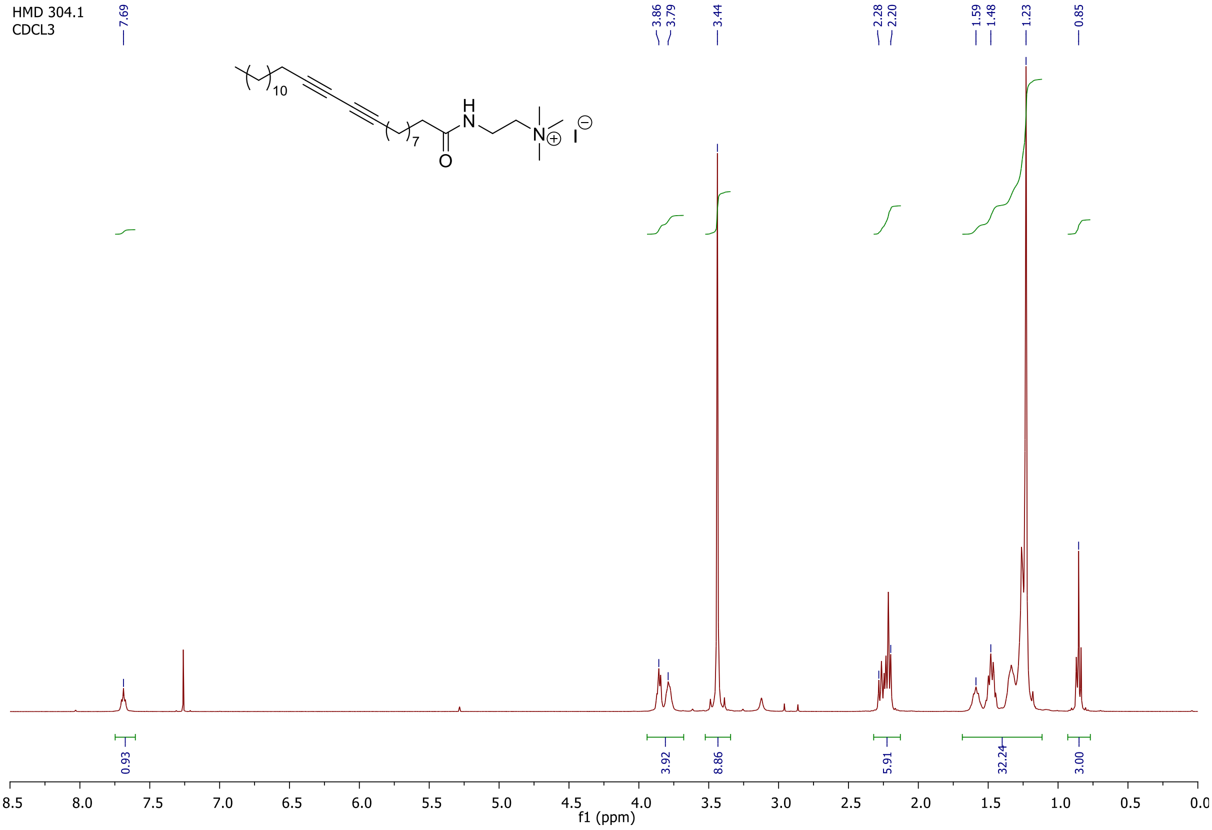

^{13}C-NMR

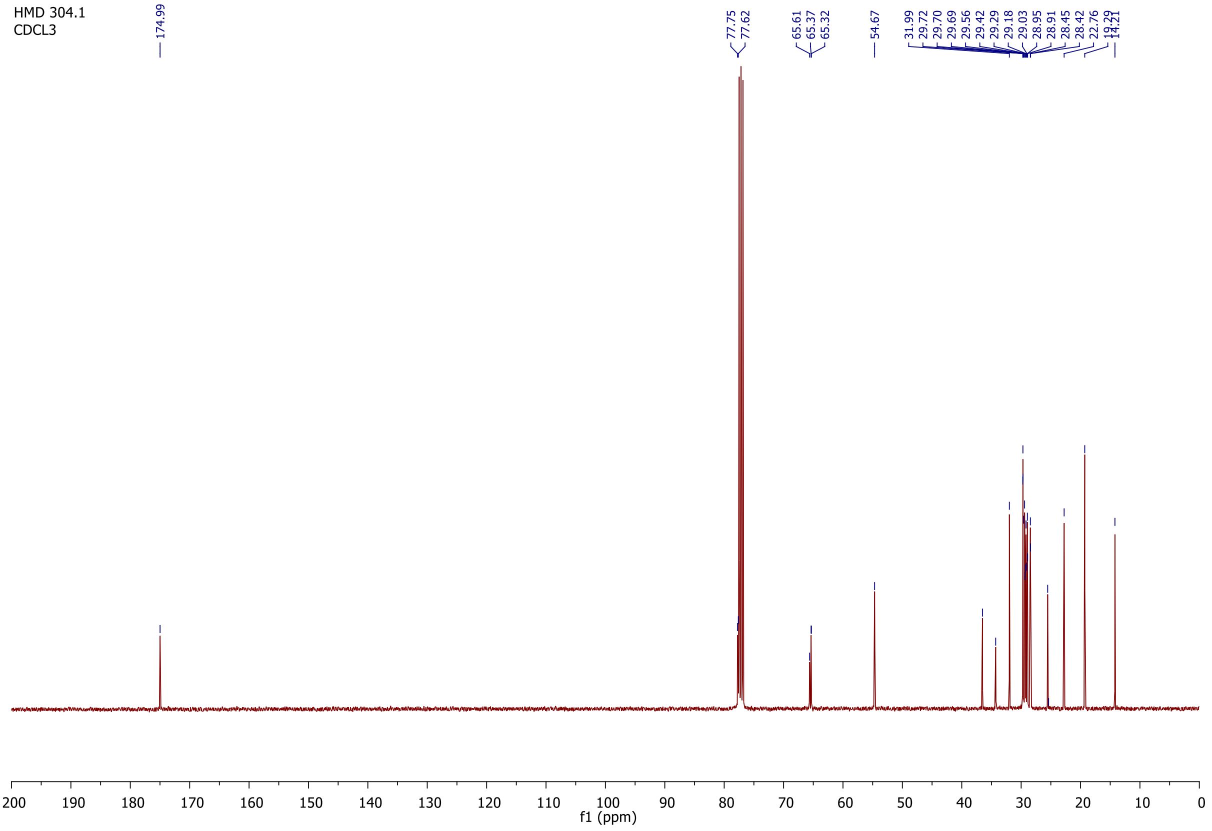

***N*,*N*,*N*-trimethyl-2-(pentacosa-10,12-diynamido)ethan-1-aminium chloride (4)**

^{1}H-NMR

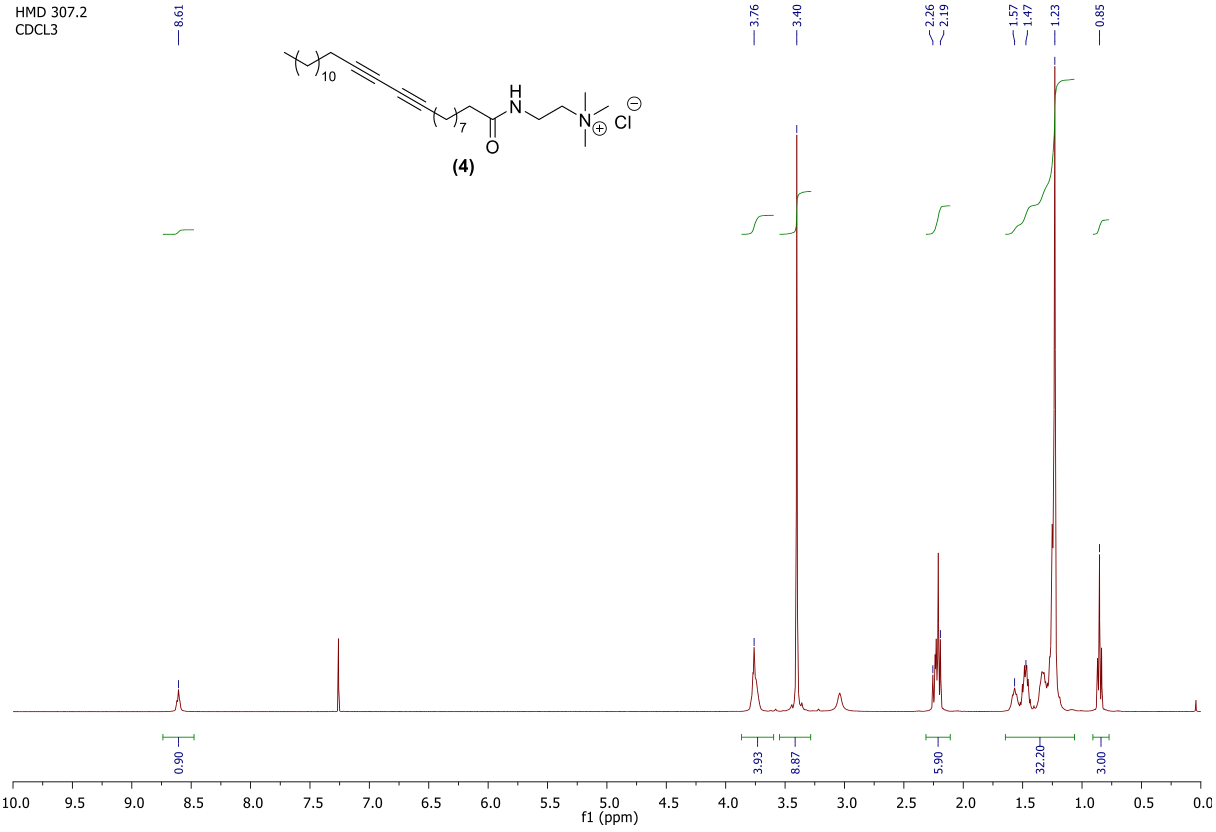

^{13}C-NMR

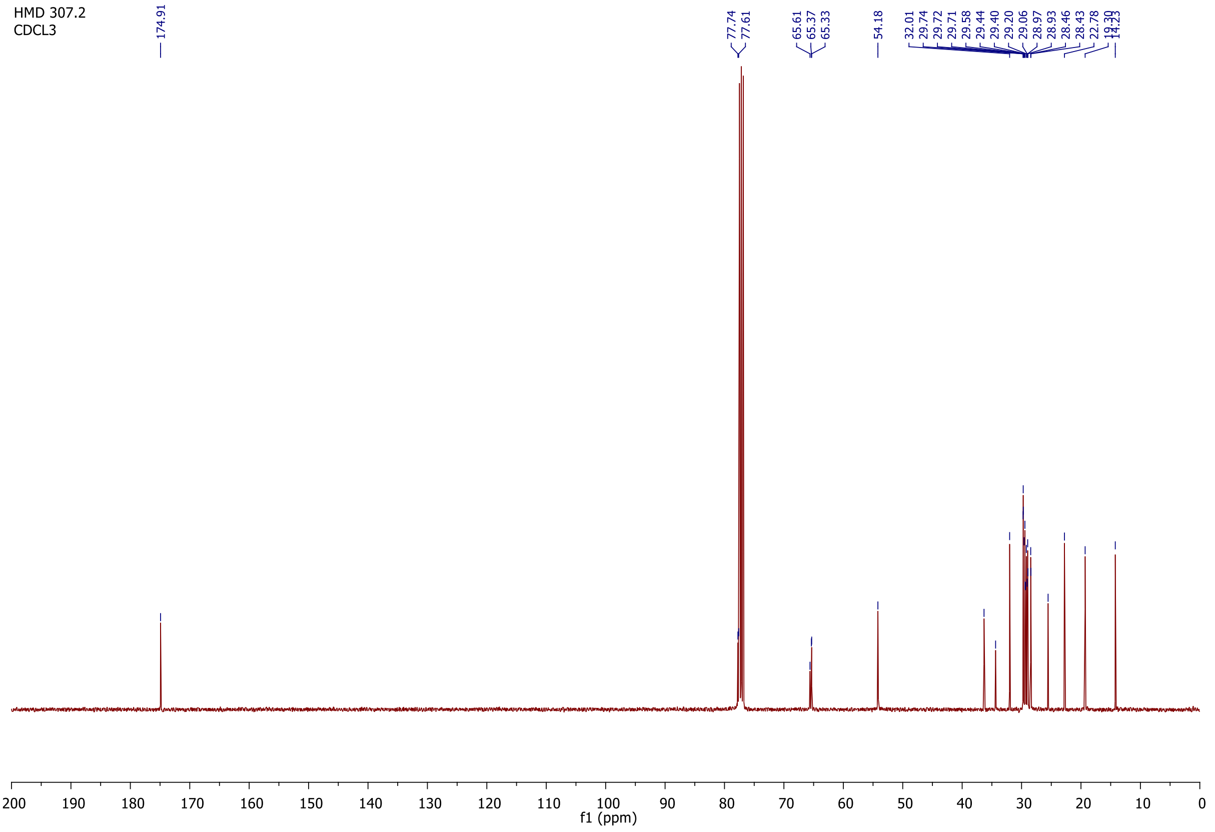

6- Cytotoxicity studies of micelles

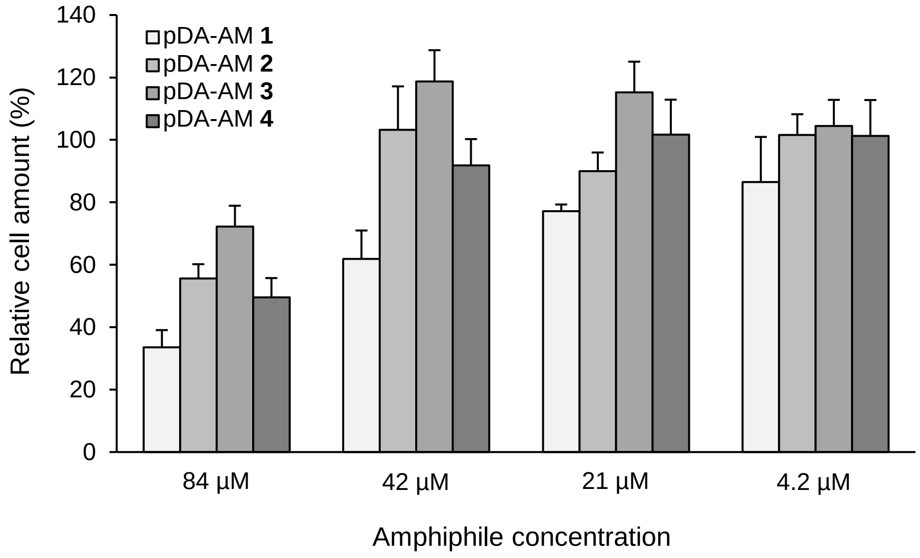

Figure S1: Cell proliferation/survival assay of Hela cells exposed to increasing concentrations of pDA-AM **1**, pDA-AM **2**, pDA-AM **3**, and pDA-AM **4** micelles. 72 h post exposure to the micelles, cells were fixed, nuclei were stained with Hoechst 33342, and the cell amounts into culture wells were assessed by High Content imaging. Results are expressed as the average relative cell amount in treated wells normalized to untreated wells.

7- Dynamic Light Scattering of micelles

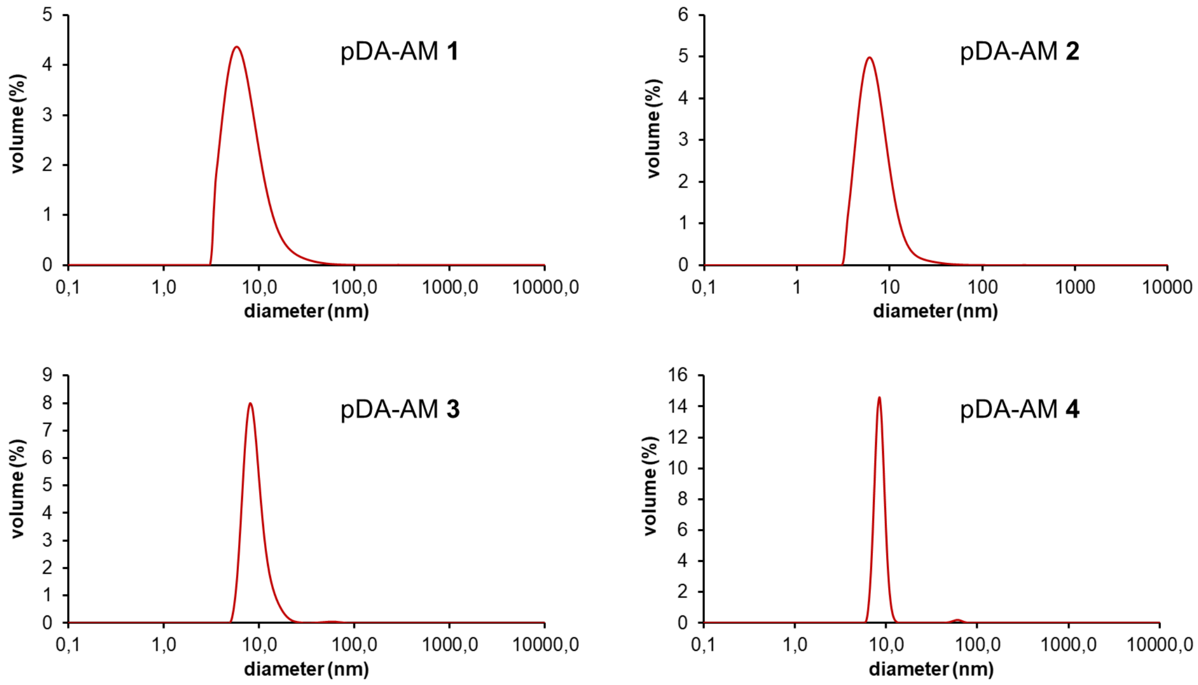

Figure S2. DLS profiles of the different micelle types.

8- DLS and zeta potential of siRNA@pDA-AM 2

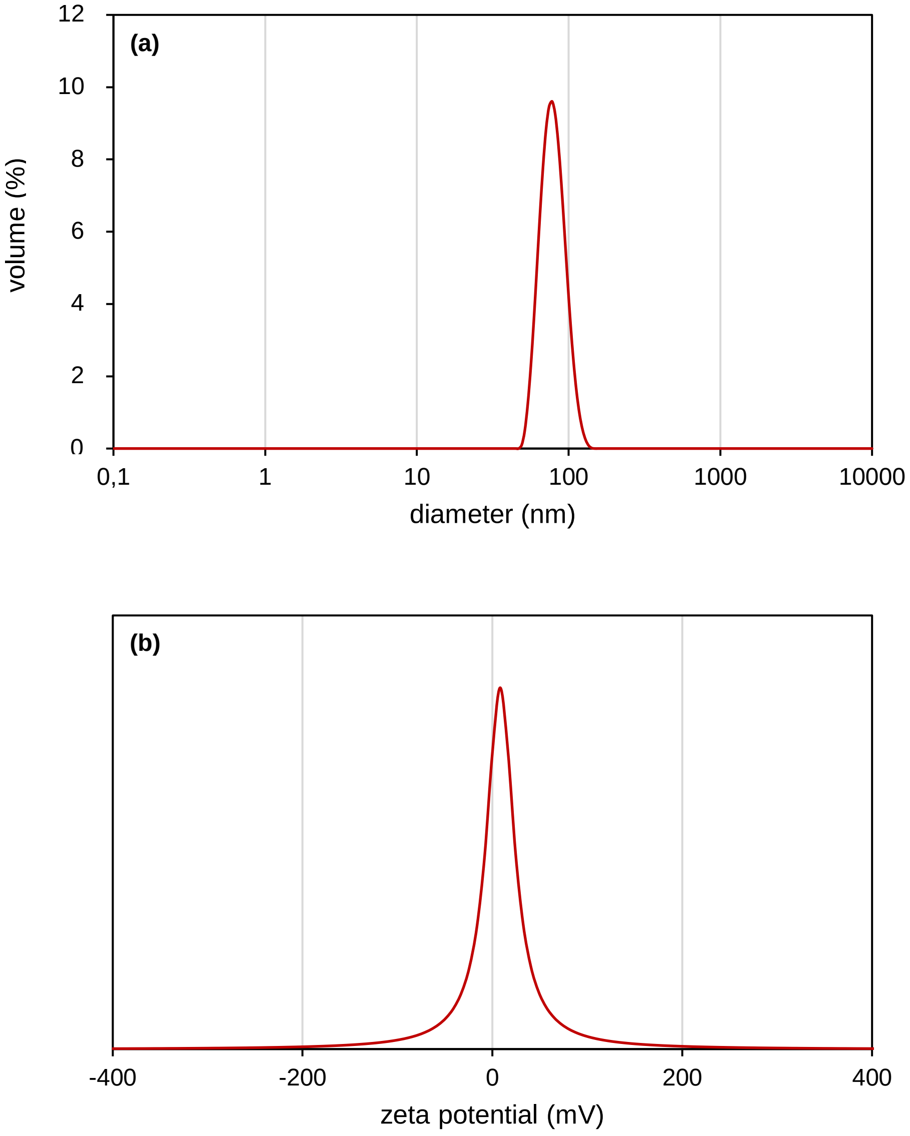

Figure S3: (a) DLS and (b) zeta potential profile of pDA-AM **2** micelles-siRNA. Complexes were prepared by mixing 143 µL of pDA-AM **2** micelles (7 mg mL^{-1}) with 250 µL of siRNA (20 µM) (N/P = 10) in a total volume adjusted to 1 mL with Hepes buffered glucose (pH 7.5). Recordings were performed after 20 minutes of incubation at room temperature.

9- Epifluorescence images of transfected HeLa cells

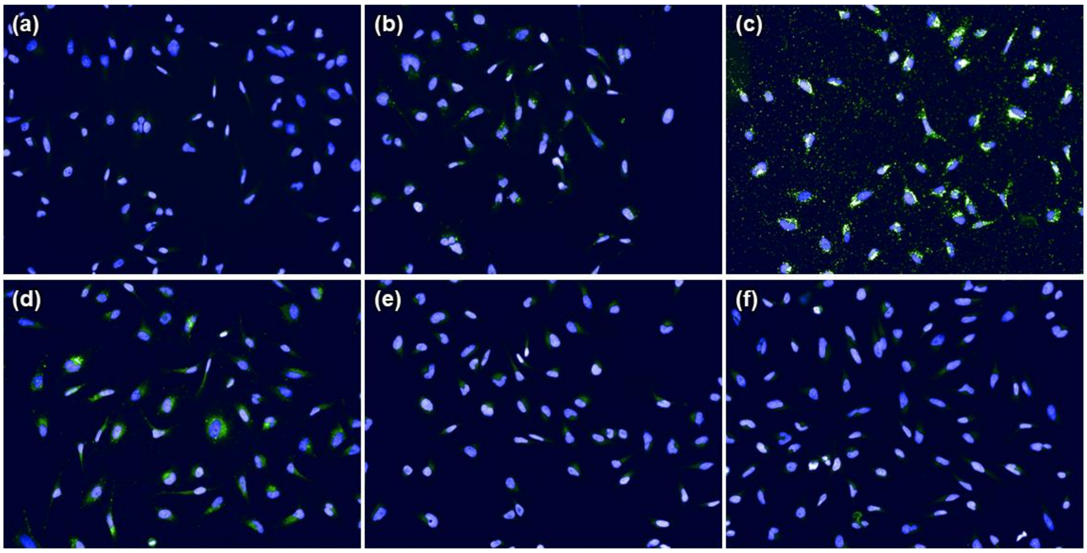

Figure S4: Representative epi-fluorescence microscopy images of: (a) untreated HeLa cells, (b) HeLa cells transfected with siRNA-Alexa 488 (green color) in complex with Lipofectamine RNAi Max, (c) pDA-AM **1**, (d) pDA-AM **2**, (e) pDA-AM **3**, (f) pDA-AM **4**. Final siRNA concentration set at 10 nM, and N/P ratios at 10. Nucleus staining was performed with Hoechst 33 342 (blue color).

10- Confocal Fluorescence images of transfected HeLa cells

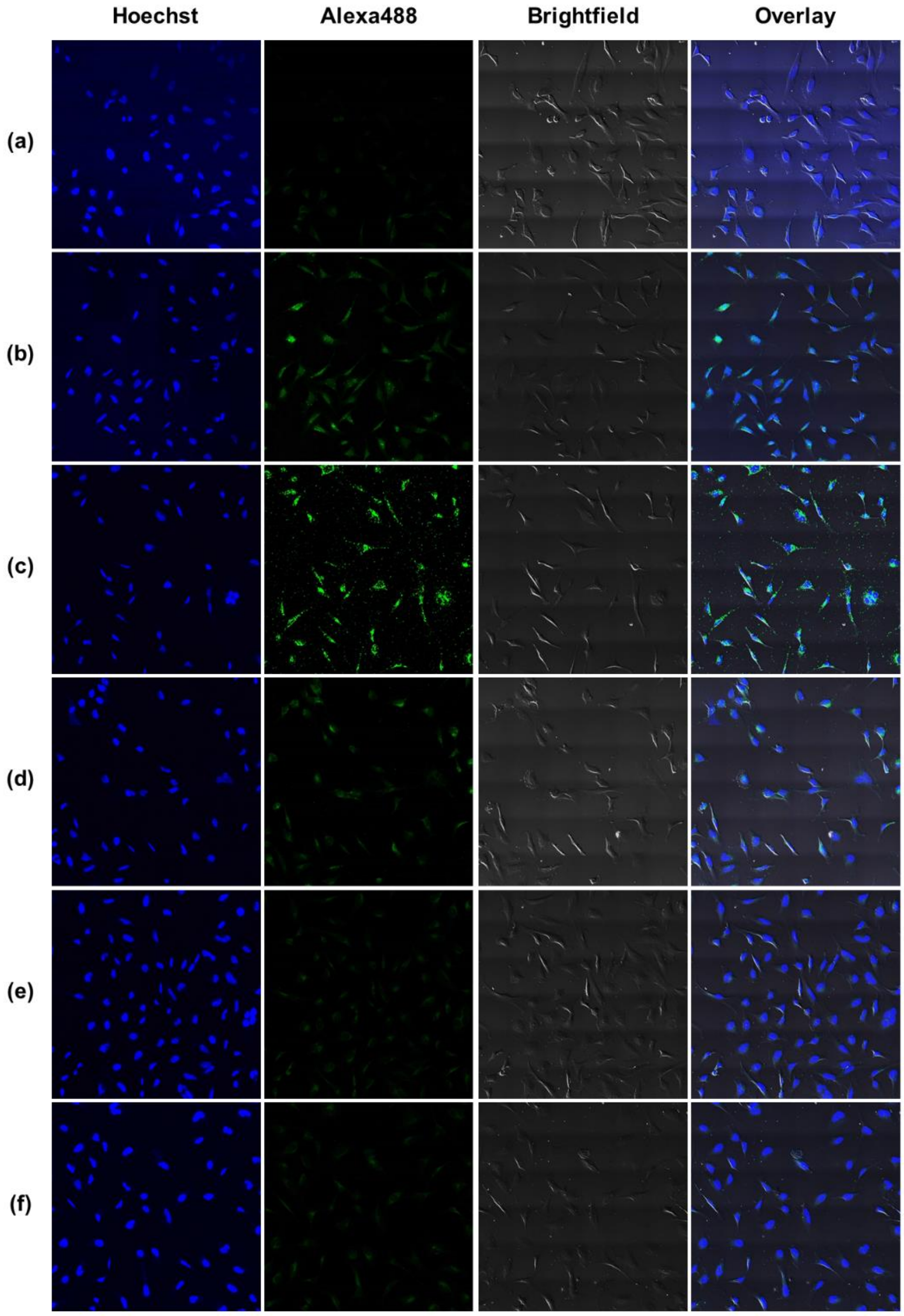

Figure S5: Confocal fluorescence images of: (a) untreated HeLa cells, HeLa cells transfected with siRNA-Alexa 488 (green color) in complex with (b) Lipofectamine RNAi Max, (c) pDA-AM **1**, (d) pDA-AM **2**, (e) pDA-AM **3** or (f) pDA-AM **4**. Final siRNA concentration set at 10nM, and N/P ratios at 10. Nucleus staining was performed with Hoechst 33 342 (blue color).
